# Genome-wide identification and evolution of the tubulin gene family in *Camelina sativa*

**DOI:** 10.1186/s12864-024-10503-y

**Published:** 2024-06-14

**Authors:** Rostyslav Y. Blume, Anastasiia M. Rabokon, Mykola Pydiura, Alla I. Yemets, Yaroslav V. Pirko, Yaroslav B. Blume

**Affiliations:** 1 Institute of Food Biotechnology and Genomics of National Academy of Sciences of Ukraine, Kyiv, 02000 Ukraine; 2 JSC “Farmak”, Kyiv, 04080 Ukraine

**Keywords:** Ancestral Crucifer Karyotype, *Camelina sativa*, Cytoskeleton, Gene evolution, Polyploidy, Tubulins

## Abstract

**Background:**

Tubulins play crucial roles in numerous fundamental processes of plant development. In flowering plants, tubulins are grouped into α-, β- and γ-subfamilies, while α- and β-tubulins possess a large isotype diversity and gene number variations among different species. This circumstance leads to insufficient recognition of orthologous isotypes and significantly complicates extrapolation of obtained experimental results, and brings difficulties for the identification of particular tubulin isotype function. The aim of this research is to identify and characterize tubulins of an emerging biofuel crop *Camelina sativa*.

**Results:**

We report comprehensive identification and characterization of tubulin gene family in *C. sativa*, including analyses of exon-intron organization, duplicated genes comparison, proper isotype designation, phylogenetic analysis, and expression patterns in different tissues. 17 α-, 34 β- and 6 γ-tubulin genes were identified and assigned to a particular isotype. Recognition of orthologous tubulin isotypes was cross-referred, involving data of phylogeny, synteny analyses and genes allocation on reconstructed genomic blocks of Ancestral Crucifer Karyotype. An investigation of expression patterns of tubulin homeologs revealed the predominant role of N^6^ (A) and N^7^ (B) subgenomes in tubulin expression at various developmental stages, contrarily to general the dominance of transcripts of H^7^ (C) subgenome.

**Conclusions:**

For the first time a complete set of tubulin gene family members was identified and characterized for allohexaploid *C. sativa* species. The study demonstrates the comprehensive approach of precise inferring gene orthology. The applied technique allowed not only identifying *C. sativa* tubulin orthologs in model *Arabidopsis* species and tracking tubulin gene evolution, but also uncovered that *A. thaliana* is missing orthologs for several particular isotypes of α- and β-tubulins.

**Supplementary Information:**

The online version contains supplementary material available at 10.1186/s12864-024-10503-y.

## Introduction

Cell division is extremely important for the growth, development, and reproduction of any eukaryotic organism represents. One of the principal cytoskeleton systems, participating in cell division, are microtubules, consisting of polymerized tubulins [1, 2]. Tubulins may be considered one of the most conserved proteins in eukaryotes and they are encoded by multiple gene families in plants [3–6]. The vast majority of tubulins (more than 80%) are represented by α- and β-tubulins gene families [7–11]. Having similar three-dimensional structures, they can form dimers, which serve as basic subunits of microtubules [1, 12, 13]. Later γ-tubulin has been discovered in plants [14]. γ-tubulin is located in different types of plant microtubule-organizing centers and plays an important role not only in microtubule formation, but also in cell division regulation [15–18].

Plant tubulin genes tend to have tissue- and/or organ-specific expression patterns, varying on different stages of ontogenesis [7, 11, 19, 20]. Respective level of sequence diversity and differential regulation of α- and β-tubulin subfamilies may confer flexibility in cell wall formation in *Populus* [9]. α-Tubulin genes are differently expressed during cold acclimation of winter and spring soft wheat (*Triticum aestivum*) [21] and sugarcane (*Saccharum* spp. hybrids) [22]. It was shown that seven of the nine *GhTUA* genes are predominantly expressed in developing fibers of cotton (*Gossypium hirsutum*). Among them, *GhTUA9* displayed the highest level of expression, revealing its fiber specificity [23]. Histochemical assays demonstrated that the *GhTUA9:GUS* gene was specifically expressed in elongating fibers. Recently, three candidate tubulin genes were identified in upland cotton which may play a key role during fiber development complementing fiber length and strength [24]. For instance, the α-tubulin gene *Tuba1* distinctively presented in a cytoplasmic male sterile and its maintainer lines of non-heading Chinese cabbage (*Brassica rapa*), demonstrating that this gene played an important role in the development of pollen and may be closely related to male sterility [25].

Tubulin gene mutations may give rise to abnormal plant growth and development [26]. For example, the decreased expression of α-tubulin 6 (TUA6) gene in *Arabidopsis thaliana* led to abnormal cell division in shoot tips and inhibited root elongation [27]. Overexpression of cotton *GhTUA9* in fission yeast (*Schizosaccharomyces pombe*) promoted atypical longitudinal growth of the host cells by 1.4–1.7-fold, indicating that the *GhTUA9* gene is involved in cell elongation [23]. Transgenic rice plants with antisense expression of β-tubulin 8 (OsTUB8) were inhibited in the amount of seed set after ripening, and the height of plants was 20–60% lower in comparison with wild type [28].

In experiments with quantitative real-time (RT) PCR analysis in willow (*Salix arbutifolia*), it was shown that the small number of TUA gene family members relative to that of TUBs (eight TUA against twenty TUB genes) was complemented by a higher transcript copy number for each TUA gene, which is essential to the maintenance of the tubulin 1:1 heterodimer assembly [29]. Analysis of α-tubulin genes in intraspecific taxa of *Miscanthus sinensis* and its close relatives *M. floridulus* and *M. condensatus* demonstrated wide occurrence of the trans-species polymorphisms in these genes and the approximately equal frequency of each allelic type what makes it extremely unlikely that α-tubulin diversity has been maintained under neutrality [30]. Respectively, balancing selection may have contributed to such an apportioning of genetic variability as well as to high levels of genetic variation in α-tubulin of *M. sinensis* [30]. In the same way, the results of whole-genome identification and comprehensive analyses of the phylogeny and synteny of the β-tubulin genes of interspecific hybrids between peach (*Prunus persica*) and almond (*Prunus dulcis*) can be interpreted [31].

Duplicated tubulin genes in polyploids could be disrupted or may undergo subfunctionalization, diversifying in expression patterns as it was demonstrated for *Malus domestica*, in which the vast majority of duplicated genes showed uncorrelated expression profiles, including tubulin genes [32]. Similarly retained α-tubulin genes in mesopolyploid *B. rapa* possessed distinct expression patterns and showed different organization of promoter regions [33], suggesting that these highly similar conserved genes have faced subfunctionalization after whole genome duplication (WGD) event.

Being widely represented among *Brassicaceae* family which contains about 338 genera and more than 3700 species around of the world, the polyploids include many common oilseed crops [34]. Some of them rapidly emerged as important crops or the model system for plant biology including *Arabidopsis* belonging to the tribe Camelineae and the allohexaploid *Camelina sativa* (L.) Crantz. (2*n* = 40), also known as false or wild flax, German sesame, gold-of-pleasure, or linseed dodder [35–37]. This interest was restored due to biofuel or jet fuel potential of the oil from the seeds of *Camelina* [38–41]. It was reported that *Camelina* oil can serve as a feedstock for jet biofuel which is characterized by significantly lower greenhouse gas emission than petroleum-based jet fuel (by up to 75%) [42]. In addition to its use for biofuel production, a broad range of nutritional, medicinal and industrial applications of the oil have been described [35, 36, 38, 39, 43, 44]. *Camelina* oil is characterized by a high content of unsaturated fatty acids (more than 90%), low concentration of erucic acid and high levels of natural antioxidants (tocopherols) [36, 45].

Several types of bacteria and almost all types of eukaryotic cells have the capability to accumulate lipids, with the mechanism of lipid storage being fairly conserved from yeast to mammals and plants [46]. Fatty acids released from cellular membranes are stored as triacylglycerols in lipid droplets. It was demonstrated that microtubules and tubulin in particular participate actively in the and transport of lipid droplets in plants [47, 48]. Because this mechanism is very deeply involved in the lipid accumulation in oilseed crops [49], it can be supposed that tubulin and especially some specific its isotypes could specify interaction between microtubules and lipid droplets.

The sets of genes of the β-tubulin subfamily were identified, which, however, was complicated by the polyploid nature of *C. sativa* and the conservancy of highly similar (highly identical) tubulin genes [50]. Taking into account that a genome draft of *C. sativa* has been published and the sequence analysis confirmed allohexaploid genome structure of this species [51], we analyzed the gene characteristics, phylogeny, gene structure, gene repetition, and expression profiles in various tissues, of all tubulin gene family members in *C. sativa*.

## Materials and methods

### Initial identification and analysis of tubulin genes in the genome of C. Sativa

Initial search for tubulin genes in the genome of *C. sativa* was conducted via BLAST (https://blast.ncbi.nlm.nih.gov/Blast.cgi) searches against the genome assembly release GCA_000633955.1, which is deposited in NCBI database and considered as the reference *C. sativa* (cv. DH55) genome [51]. The TUA1–3, TUB1–9 та TUG1-2 protein sequences of *A. thaliana* [52] and *AlTUB* protein in *A. lyrata* were used as queries for TBLASTN searches using the E-value of 1e^− 5^, comparison matrix of BLOSUM62, word length of 3. We have analyzed the results and discarded short and non-meaningful hits. The identified *C. sativa* tubulin proteins were used for additional searches against the reference *C. sativa* genome too. Subsequently, these identified sequences were analyzed for the presence of conserved tubulin-specific domains using the InterProScan (https://www.ebi.ac.uk/interpro/) tool [53]. Further, HMMER software with default parameters and conserved tubulin domains was used to search for the mentioned sequences [54].

Information on the genes in *C. sativa*, including location, genomic coordinates, sequence ID, genomic sequence, protein sequence, and coding sequence (CDS), was obtained from the NCBI database. Draft information on the orthologous hits of the identified genes was retrieved from KEGG Genome database (https://www.genome.jp/genome/), as well as information on the orthology of genes, flanking the identified tubulins. Locus ID (in CsaXXgXXXXX format) was determined via EnsemblPlants database (http://plants.ensembl.org) search using genomic coordinates of particular gene as a query. Further, these loci IDs were used to verify presence/absence of tubulin genes on homologous chromosomes.

Genomic landscape/context of the identified tubulin genes was investigated using NCBI Genome Data Viewer (https://www.ncbi.nlm.nih.gov/genome/gdv/). In order to designate correctly the isotypes of α-, β-, γ-tubulins, encoded by a particular gene, we have investigated the similarity of genomic context of potentially orthologous tubulin genes in genomes of different species. This approach was used in the cases when sequence identity data was not enough to identify real orthologues of *C. sativa* highly similar tubulin genes in genomes of other diploid species. Two *Arabidopsis* species were used as referent diploid genomes: *A. thaliana*, which appears to be one of the basal Camelineae species [55]; and *A. lyrata*, since its genome possesses the closest genome structure to ACK [56]. Additionally, two reference genomes outside the Camelineae tribe, *Alyssum linifolium* (Phytozome genome ID: 472, basal Brassicaceae species) and *Descurainia sophioides* (ID: 482, representative of Lineage I) were selected. Search for homologues of particular tubulin isotypes within genomes of these species, as well as investigation of the genomic landscape of tubulin loci was conducted using the Phytozome v13 database (https://phytozome-next.jgi.doe.gov/) via the procedure, similar to the described above.

### Gene and peptide structure analysis

Exon-intron structures of tubulin genes were derived using the Gene Structure Display Server (http://gsds.cbi.pku.edu.cn) [57]. Presence of common conserved motif among the identified tubulin peptides was analyzed using MEME Suite 5.5.2 (https://meme-suite.org/meme/index.html) tool [58]. Domain organization of the identified tubulin peptides was analyzed using InterPro (https://www.ebi.ac.uk/interpro) tool [54]. Peptide sequences were searched against the database of functional domains (Pfam) [59] and database of structural domains (CATH-Gene3D) [60]. Identified peptide motifs and domains were visualized using TBtools v2.045 software [61].

### Genomic blocks reconstruction and synteny analysis

The identified tubulin genes were visualized on *C. sativa* chromosomes using MapChart 2.32 [62], basing on the genomic coordinates of 5`-end of respective genes. Chromosomes were grouped according to subgenome, to which they belong. To do that recent data on *C. sativa* subgenome differentiation was used (Table 1). Letters A, B or C were assigned to tubulin gene names in order to show their affiliation to particular subgenome.

**Table 1:** Subgenomes of *C. sativa* and their possible progenitors [37, 63, 64]

Subgenome 1 (N, or N^6^ genome) A genome	Subgenome 2 (N, or N^7^ genome) B genome	Subgenome 3 (H, or H^7^ genome) C genome
*C. neglecta* (*n* = 6)	*C. neglecta*’s ancestor (*n* = 7)	*C. hispida* (*n* = 7)
Csa04, Csa07, Csa08, Csa11, Csa14, Csa19	Csa01, Csa03, Csa06, Csa10, Csa13, Csa16, Csa18	Csa02, Csa05, Csa09, Csa12, Csa15, Csa17, Csa20

Further, the location of ACK genomic blocks within camelina chromosomes was reconstructed in order to clarify nature of tubulin genes homology (orthology, paralogy, homeology, etc.) as well as the nature of their pseudogenes. To do that, we followed the available data on *A. thaliana*-*C. sativa* genomes synteny [51], which included the total list of syntelogous gene pairs and their affiliations to particular genomic blocks. Using this dataset, we have retrieved coordinates for genomic block borders and merged them with tubulin location data. Allocation of the identified genes together with ACK blocks was visualized using MapChart 2.32. The reconstruction of ACK blocks and tubulins genes allocation was performed for *A. thaliana* and *A. lyrata* species via similar procedure using previously published ACK location data for these species [65–67].

Syntenic relations between homoelogous tubulin genes from different *C. sativa* subgenomes were analyzed in TBtools v2.045 software [61], using MCScanX algorithm [68]. The results were further visualized as circos plot. Together with ACK blocks reconstruction data both analyses allowed segregating homeologous and paralogous genes. To explore the synteny relationships of the orthologous tubulin genes of *C. sativa* with *Arabidopsis* species, the genome data and the gene annotation files of *A. thaliana* (TAIR annotation release 10) and *A. lyrata* were downloaded from NCBI database. The synteny analyzing dual plots graphs were constructed by using the Dual Synteny Plotter function in TBtools, while inter-genome synteny was inferred using MCScanX algorithm also.

Accounting the identified gene homeologs relations and their orthologs with tubulin gene family members in *A. thaliana* and *A. lyrata*, *Ka* and *Ks* values were calculated using built in function in TBtools v2.045 software [61].

### Phylogenetic analysis

The peptide sequences of the tubulin genes identified in this study and the previously reported sequences of α-, β- and γ-tubulins from other plant species (Tables S2, S4, S6) were used. Respective amino acid sequences of tubulins were aligned using MUSCLE algorithm [69]. Initial isotype determination of tubulin proteins was performed on the basis of the Neighborhood Joining (NJ) tree construction results, performed in MEGAX [70].

Additionally, the optimal substitution models for Maximum Likelihood (ML) tree inference were identified using ModelFinder [71]. For the sets of α- and β-tubulins JTT + F + I + G6 was determined as the optimal, and for γ-tubulins – JTT + I + G4 as the best model. Phylogenetic analysis (ML) was performed using web version of IQ-TREE tool (http://iqtree.cibiv.univie.ac.at) [72, 73] with the bootstrap support of 1000 iterations, involving the usage of UFBoot for ultrafast bootstrapping [74]. The resulting trees were visualized using the web-version of iTOL v6 tool (https://itol.embl.de) [75]. Trees were rooted to the respective node of *Chlamydomonas reinhardtii* protein, which was used as the outgroup. Classes of α- and β-tubulins were identified on the basis of the performed phylogenetic analysis results, which were compared with previously published ones [29].

### Expression analysis

The transcriptomics data of *C. sativa* (cv. DH55) were obtained from the publicly available database [76]. The expression levels of the identified tubulin genes in twelve different tissues (at different developmental stages) were taken for the analysis. Tubulins expression was analyzed in following tissues: germinating seed (GS), cotyledon (C), young leaf (YL), senescing leaf (SL), stem (S), root (R), flower bud (B), flower (F) and seeds/fruits at various developmental stages – early (ESD), early-mid (EMSD), late-mid (LMSD) and late (LSD). Expression heatmap was constructed using Heatmapper tool (http://heatmapper.ca) [77]. Clustering method was Average Linkage with Euclidean distances. Additionally, downregulation of tubulin genes under salt stress conditions was analyzed based on the specific *C. sativa* transcriptome database, constructed using the same referent cultivar DH55 [78].

## Results

### Identification and characterization of α-tubulins

Initially, 17 genes of different α-tubulin isotypes were identified, while 15 encoded fully functional proteins. One gene, *CsTUA1-Un*, encoded 444 a.a. protein, which is 6–7 a.a. shorter than the regular α-tubulin, while *CsTUA5p-A* appeared to be a pseudogene of *TUA5* (Table 2). Commonly, *CsTUA* genes encode putative protein of 450 a.a. However, three homeologs *CsTUA4-A*, *CsTUA4-B*, and *CsTUA4-C* encoded 451 a.a. peptide, because of 442-443insE. Interestingly, either *A. thaliana* or *A. lyrata* orthologs of these *TUA4* genes do not encode 451 a.a. peptide and, thus, do not have glutamate residue at this position. *CsTUA1-Un*, which is the only tubulin gene unassigned to chromosome, encodes slightly shortened peptide, due to deletion of SVFEPS region, located at 294–299 position. The pseudogene *CsTUA5p-A* is comprised of fragments of two 5`-terminal exons of regular *CsTUA5-B*/*C* and encodes 183 a.a.-long C-terminal fragment of regular TUA5 protein.

**Table 2:** The identified α-tubulin genes in the genome of *C. sativa*

Proposed gene name	NCBI gene ID	Gene length (bp)	Exons	Peptide length (aa)	*A. thaliana* / *A. lyrata* isotype	TUA class	Chr, sub-genome
*CsTUA2-A*	104,742,538	2266	4	450	TUA2/4/6	I	14, G1
*CsTUA2-B*	104,777,925	2266	4	450	TUA2/4/6	I	3, G2
*CsTUA2-C*	104,758,270	2138	4	450	TUA2/4/6	I	17, G3
*CsTUA4-A*	104,765,838	2219	4	451	TUA2/4/6	I	19, G1
*CsTUA4-B*	104,784,567	2193	4	451	TUA2/4/6	I	1, G2
*CsTUA4-C*	104,746,364	2246	4	451	TUA2/4/6	I	15, G3
*CsTUA6-A*	104,703,779	2267	4	450	TUA2/4/6	I	7, G1
*CsTUA6-B*	104,752,837	8668	4	450	TUA2/4/6	I	16, G2
*CsTUA6-C*	104,714,511	2598	4	450	TUA2/4/6	I	9, G3
*CsTUA1-A*	104,700,952	2319	5	450	TUA1	II	7, G1
*CsTUA1-Un*	104,773,561	5961	5	444	TUA1	II	Scaff00574
*CsTUA3-A*	104,706,313	2150	5	450	TUA3	II	8, G1
*CsTUA3-B*	104,736,043	2041	5	450	TUA3	II	13, G2
*CsTUA3-C*	104,770,349	2079	5	450	TUA3	II	20, G3
*CsTUA5p-A* ^*^	104,706,312	840	2	--	TUA5*	II	8, G1
*CsTUA5-B*	104,736,047	2114	5	450	TUA5	II	13, G2
*CsTUA5-C*	104,770,351	2052	5	450	TUA5	II	20, G3

*- pseudogene

Initially, we performed attempts to differentiate α-tubulin genes, based on the alignment of their CDS or brief phylogenetic analysis (at the scale of *C. sativa*, *A. thaliana* and *A. lyrata*) of their encoded protein product. However, such approaches allowed distinguishing these genes only at the level of α-tubulin Classes I and II (Fig. S1). Using such approach only CsTUA1 proteins of all three species were successfully distinguished as the members of separate isotype inside the Class II (Fig. S1). Additional recovery of conserved motifs and domains distributed within the identified α-tubulin peptides appeared to be non-informative for isotype differentiation (Fig. S2). Almost all α-tubulins possessed the presence of same 11 conserved motifs, except for *CsTUA1-Un*, which had three distinct motifs, associated with the mentioned SVFEPS region deletion (Fig. S2a). Respectively, all α-tubulin peptides tend to have two functional domains (GTPase and Tubulin C-terminal domains), if Pfam database is used as a reference (Fig. S2b); or 3 domains (GTPase, Tubulin C-terminal and C-terminal tail domains), if Gene3D database is used as a reference (Fig. S2c). Therefore, further isotype differentiation of TUA2, TUA4 and TUA6 genes, as well as TUA3 and TUA5 genes was made basing on their loci differences (described in details in Supplementary Note 1 and Table S1). Due to the extremely high level of sequence conservancy of α-tubulins investigated here, isotypes were referred as orthologous lineages of the genes (e.g. TUA3 and TUA5 genes could be successfully differentiated in *C. sativa*, despite they encode extremely similar peptides (99.56–100%). Later, the described results of loci differentiation were confirmed by the results of synteny analysis, mentioned below.

The exon-intron structures of the identified *C. sativa TUA* genes were analyzed (Fig. S3). TUA genes have conserved, but more diverse exon-intron structures than other tubulin genes. Number of exons of *TUA* genes is different within different TUA Classes. For example, *CsTUA2-A*/*B*/*C*, *CsTUA4-A*/*B*/*C* and *CsTUA6-A*/*B*/*C* genes all have four exons, what is typical for representatives of TUA Class I [29]. On the other hand, all representatives of TUA Class II (*CsTUA1-A*/*Un*, *CsTUA3-A*/*B*/*C* and *CsTUA5-B*/*C*) have five exons. No exceptions from the general rule of *TUA* exon-intron structure were observed. However, intron length varied even within homeologous genes of a particular isotype. The most significant difference was observed in length of second introns of *CsTUA1-A* and *CsTUA1-Un*. In the case of *CsTUA1-A* length of the second intron was 315 bp, whereas for *CsTUA1-Un* it was 3912 bp.

Three α-tubulin genes *CsTUA6-B*, *CsTUA6-C* and *CsTUA1-Un* possess alternative splice variants. *CsTUA6-B* and *CsTUA6-C* have alterations in the transcription of fourth exon. For both genes it can be transcribed partially, while the missing part of CDS will be compensated by part of UTR, which will be expressed as a part of the fifth exon in such case. The five-exon variant of *CsTUA6-B* is characterized by the biggest spliced intron (fourth intron that arise only for five-exon variant), which is 6427 bp long. *CsTUA1-Un* does not possess any alteration in exon-intron structure. However, its second intron (which is the longest one among canonical TUA gene variant of *C. sativa*) encodes transposase-derived putative nuclease HARBI1 (104,773,559) might be still amenable for expression of full-length, possibly functional, peptide.

**Fig. 1:**
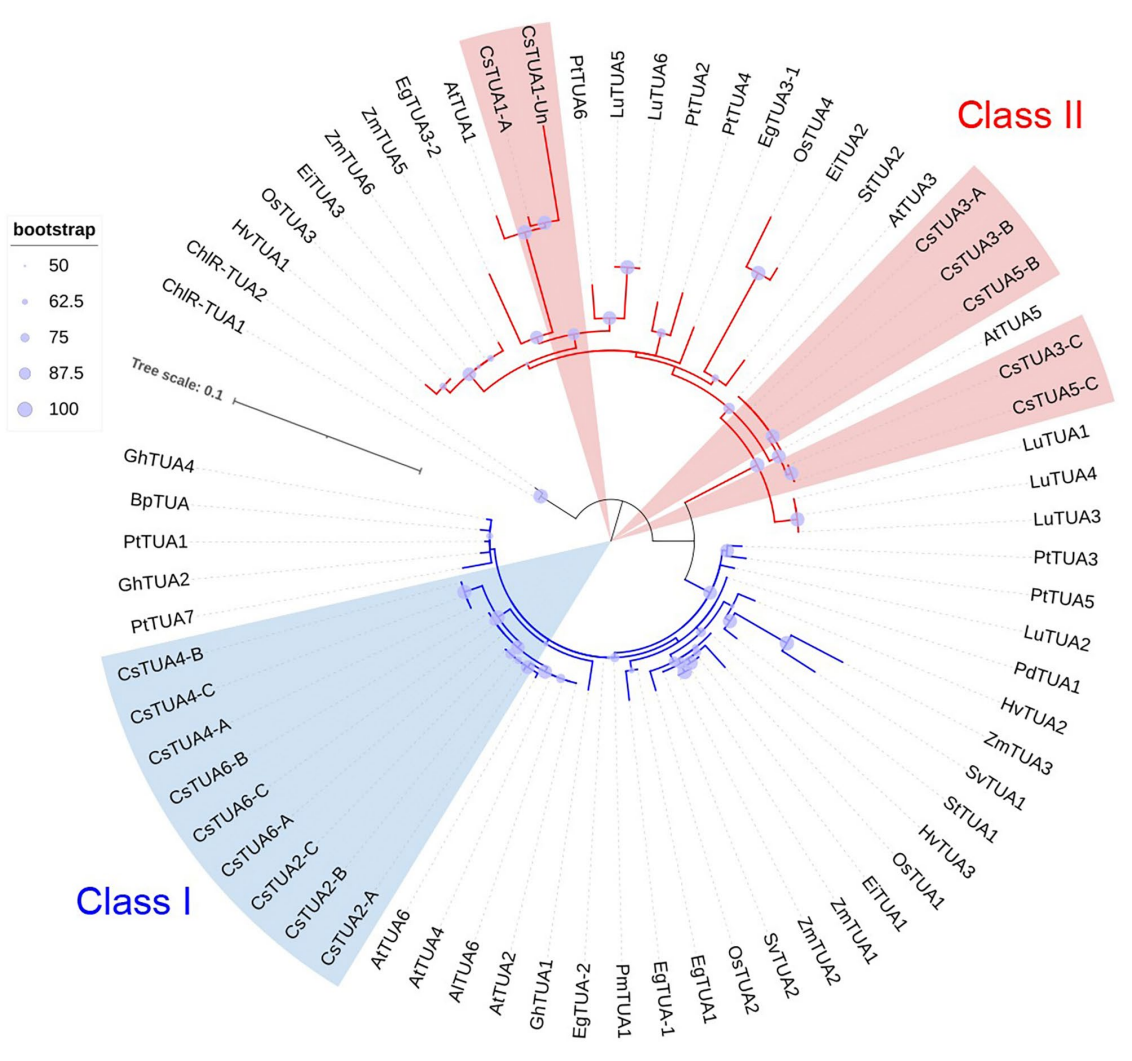
Phylogenetic analysis of plant α-tubulins. Maximum likelihood analysis was performed involving 68 proteins sequences from 17 species, the gene list is provided in Table S2. This phylogenetic tree was constructed with the bootstrap support of 1000 iterations, low bootstrap values are excluded from tree representations

A total of 68 α-tubulin protein sequences (Table S2), including 16 translated peptides of *C. sativa* genes, were aligned, and further used for phylogenetic analysis (Fig. 1). All analyzed sequences were placed into two major clades, corresponding to Class I and II of α-tubulins. The clade of Class II TUA is characterized by greater branch length, which may indicate the higher sequence diversity within Class II. Sequences of all representatives of the Camelineae tribe grouped in separate monophyletic clades with very low diversity level inside those groups (Fig. 1).

No strict separation between the dicot and monocot α-tubulins was observed inside the Class I. Monocot α-tubulins HvTUA2, ZmTUA3, SvTUA1 shared the same clade with potato α-tubulin, StTUA1. However, several dicot α-tubulins were placed as basal branches (PdTUA1, EgTUA1, etc.) in this major clade of the dicot-monocot Class I α-tubulins (Fig. 1). Brassicaceae α-tubulins (including the identified from *C. sativa* genome) were placed in their own clade inside the Class I. The proteins of other dicots were placed in various subclades inside the Class I without any specific hierarchy.

Inside the Class II monocot α-tubulins formed separate branches, often placed as the sister clades to the groups of dicot proteins (Fig. 1). The identified *C. sativa* α-tubulins were placed into different groups. CsTUA-1-A/Un were grouped together with their ortholog, AtTUA1, in a specific clade that included EgTUA3-2, PtTUA6, LuTUA5 and LuTUA6. The proteins of TUA3 and TUA5 isotypes were grouped in a separate clade, which appears to be a sister clade to almost all dicot and monocot α-tubulins of Class II. The exceptions were three linseed proteins, namely LuTUA1, LuTUA3, LuTUA4, which were basal to all representatives of Class II (Fig. 1).

### Identification and characterization of β-tubulins

The β-tubulin subfamily is being larger and more diverse than α-tubulin subfamily. A total of 26 functional β-tubulin genes and 8 pseudogenes were identified in the genome of *C. sativa* (Table 3 and S5). *TUB* genes varied in size from 1827 to 3828 bp, mostly due to the variability of the intron size. Lengths of the encoded TUB peptides appeared to be more variable than TUA ones – ranged from 444 to 451 aa, what was conditioned by C-end ‘tail’ variation in the majority of cases. TUB proteins, belonging to a particular isotype, possessed similar size or varied by 1–2 aa only. The initial identification of TUB isotypes was conducted based on their CDS alignment and a brief phylogenetic analysis (using sequences of *C. sativa*, *A. thaliana* and *A. lyrata*) of the encoded peptides, similarly to TUA (Fig. S4). This analysis allowed to distinguish the most β-tubulin isotypes with exception of almost identical TUB2 and TUB3. All other β-tubulin Classes were placed in separate clades as monophyletic groups, while Class IV was the polyphyletic group, consisting of two separate clusters of TUB1 and TUB5.

**Table 3:** The identified β-tubulin genes that encode full-length proteins

Proposed gene name	NCBI gene ID	Gene length (bp)	Putative protein length (aa)	*A. thaliana* / *A. lyrata* isotype	TUB class	Chr, sub-genome
*CsTUB6-A*	104,705,499	2440	449	TUB6	I	8, G1
*CsTUB6-B*	104,735,240	2488	449	TUB6	I	13, G2
*CsTUB6-C*	104,769,465	2401	449	TUB6	I	20, G3
*CsTUB2-A*	104,780,498	2355	449	TUB2/3	II	4, G1
*CsTUB2-B*	104,790,913	2341	450	TUB2/3	II	6, G2
*CsTUB2-C*	104,711,158	1989	450	TUB2/3	II	9, G3
*CsTUB3-A*	104,726,871	2365	450	TUB2/3	II	11, G1
*CsTUB3-B*	104,762,402	2080	451	TUB2/3	II	18, G2
*CsTUB3-C*	104,740,363	2279	451	TUB2/3	II	2, G3
*CsTUB7-A*	104,700,544	2319	449	TUB7	II	7, G1
*CsTUB7-B*	104,749,926	2242	448	TUB7	II	16, G2
*CsTUB7-C*	104,786,727	3249	449	TUB7	II	5, G3
*CsTUB8-A*	104,706,681	1901	449	TUB8	II	8, G1
*CsTUB8-B*	104,736,398	1827	449	TUB8	II	13, G2
*CsTUB4-C*	104,771,594	3032	444	TUB4	III	20, G3
*CsTUB9-A*	104,722,972	1878	444	TUB9	III	11, G1
*CsTUB9-B*	104,718,249	1926	444	TUB9	III	10, G2
*CsTUB9-C*	104,731,465	1892	444	TUB9	III	12, G3
*CsTUB1-A*	104,702,324	2521	448	TUB1	IV	7, G1
*CsTUB1-B*	104,751,460	2502	448	TUB1	IV	16, G2
*CsTUB5-A*	104,740,697	2660	449	TUB5	IV	14, G1
*CsTUB5-B*	104,776,116	3217	449	TUB5	IV	3, G2
*CsTUB5-C*	104,756,348	3828	450	TUB5	IV	17, G3
*CsTUB-A*	104,781,875	2245	446	TUB	III-like	4, G1
*CsTUB-B*	104,792,233	2427	446	TUB	III-like	6, G2
*CsTUB-C*	104,786,080	2352	446	TUB	III-like	5, G3

While isotype of the majority of β-tubulin peptides could be established by the sequence similarity, the members of TUB2 and TUB3 appear to be highly similar. Additional analysis of conserved motifs and domains of the identified β-tubulins was also non-informative for isotype differentiation (Fig. S5). Each β-tubulin has up to 12 conserved motifs (Fig. S5a), while the only variable part is C-terminal tail, which is believed to be a hypervariable domain, which is not suitable for reliable isotype differentiation [10, 19]. The distribution of functional and structural domains was also highly conserved and corresponded to three typical domains: GTPase, Tubulin C-terminal and C-terminal tail domains (Fig. S5b and Fig. S5c). Therefore, the isotypes (orthologous lineages) of TUB2 and TUB3 were investigated based on their loci differences (described in detail in Supplementary Note 2 and Table S3).

Interestingly, an additional β-tubulin isotype was identified in the genome of *C. sativa*, which was not previously found in *A. thaliana*, but present in *A. lyrata*. This isotype does not have assignment to particular number, therefore its orthologous genes/proteins in other species are named as TUB (tub-chain-like, without number) during the genome annotation. The gene of this isotype is found in *A. lyrata* (*AlTUB*, Table S3), while its three orthologs are present in all three *C. sativa* subgenomes (*CsTUB-A*/*B*/*C*). Besides high sequence identity, these genes also share a similar genomic context in *A. lyrata* and *C. sativa* species (Supplementary Note 2).

The exon-intron structure of the identified β-tubulin genes of *C. sativa* was further analyzed (Fig. S6). *CsTUB* genes possess even more conserved exon-intron structure than α-tubulin genes. Typical three-exon structure was observed for all TUB genes, regardless of their class of isotype identity. Such high conservancy of the structure of β-tubulin genes is generally observed within almost all flowering plant species, with only few exceptions: *ZmTUB1* in maize and *OsTUB2* in rice [3, 79]. In addition, a large variation of intron length was observed among the genes of different isotypes. However, the introns of homeologous genes commonly possessed similar, but not identical length. The most remarkable exceptions were *CsTUB3-B*, *CsTUB5-B*, *CsTUB5-C*, which had significantly enlarged or shortened second intron, and *CsTUB7-C*, which significantly enlarged first intron, if compared to their homeologs.

It is also worth noting the majority of β-tubulin pseudogenes arose in result of partial or complete of loss of different exons (Supplementary Note 3). The vast majority of the pseudogenes are believed to be disrupted homeologs, which were degraded after allopolyploidization of *C. sativa*. Only *CsTUB9p-A* and *CsTUB9p-B* seem to be partial duplicates of the functional *CsTUB9-A* and *CsTUB9-B* (Table S5).

**Fig. 2:**
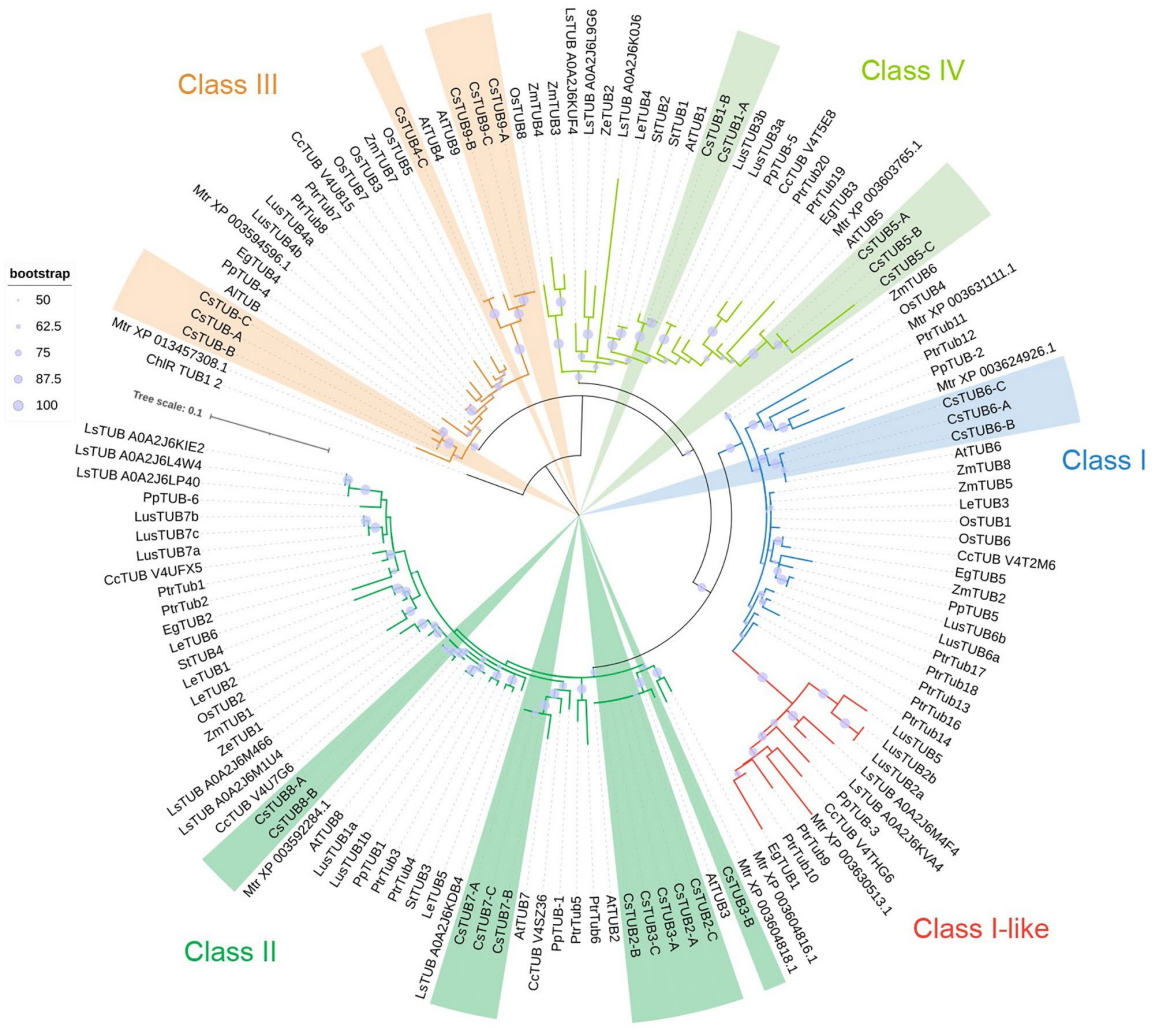
Phylogenetic relations of plant β-tubulins. Maximum likelihood analysis was performed involving 138 proteins sequences from 16 species, the gene list is provided in Table S4. The tree was constructed with the bootstrap support of 1000 iterations, low bootstrap values are excluded from tree representations

As the next stage of TUB analysis, a total of 138 β-tubulin protein sequences (Table S4), including 26 translated peptides of *C. sativa* genes, were aligned, and further used for phylogenetic analysis (Fig. 2). Analyzed β-tubulins sequences were placed into four major clades, corresponding to TUB Classes I, II, III, and IV. Unlike in the case of α-tubulins (Fig. 1), the β-tubulins of monocots were often placed into separate distinct clades (Fig. 2). The exception was the Class I, in which ZmTUB2 was grouped along with the dicot proteins.

An additional subclade was also defined as the group of specialized Class I β-tubulins, called Class I-like. Ambiguous TUB (TUB10) isotype members, absent in the genome of *A. thaliana*, but present in *C. sativa* and *A. lyrata*, were placed as the basal branch for all Class III members (Fig. 2). Our draft phylogeny reconstructions, including those provided in Figure S4 with other sets of tubulin genes have shown that the TUB10 group may be placed as a sister clade to the β-tubulins of Class III, or could be grouped together with other Classes (e.g. Class IV). Considering this ambiguity, we listed *CsTUB-A/B/C* homeologs in the Table 3 as the members of sub-Class III-like.

All major β-tubulin classes included at least one set of homologous proteins from *C. sativa*. TUB proteins of *C. sativa* were placed in clades, appropriate for their Classes, in the majority of cases in the same or sister branch with *A. thaliana* or *A. lyrata* orthologs. However, no β-tubulins of *C. sativa* were placed in Class I-like clade (Fig. 2). The Class I itself contains only the representatives of TUB6 isotype, in particular CsTUB6-A, CsTUB6-B, CsTUB6-C. At the same time, Class II appears to be a more diverse group, including the representatives of TUB2, TUB3, TUB7 and TUB8 isotypes. Moreover, the members of these isotypes formed separate subclades, except the triplets of CsTUB2-A/B/C and CsTUB3-A/B/C, which shared the same minor clade. Such grouping of TUB2 and TUB3 isotypes representatives could suggest relatively recent duplication origin of these β-tubulin groups, especially considering their extremely high sequence similarity (Fig. 2).

Class III of β-tubulins include CsTUB4-A/B/C and CsTUB9-A/B/C proteins, which share a common clade (Fig. 2). This may suggest ancient ohnologous nature of TUB4 and TUB9. The triplet of CsTUB10-A/B/C proteins was placed separately from the members of TUB4 and TUB9 isotypes, indicating their earlier speciation. The β-tubulins (CsTUB1-A/B/C and CsTUB5-A/B/C) of the Class IV were placed into distinct clades apart from each other (Fig. 2).

### Identification and characterization of γ-tubulins

The γ-tubulin subfamily appeared to be smaller than α- and β-tubulin. Six functional γ-tubulin genes and no pseudogenes were identified in the genome of *C. sativa* (Table 4). The TUG isotypes was identified based on the brief phylogenetic analysis using sequences of *C. sativa*, *A. thaliana* and *A. lyrata* (Figure S7). It allowed distinguishing the genes encoding TUG1 and TUG2 isotypes. Sequence diversity among γ-tubulins appears to be significantly lower, compared to proteins of α- and β-subfamilies. Both TUG1 and TUG2 proteins possessed the same length of 474 amino acid residues.

**Table 4:** The identified -tubulin genes that encode full-length proteins

Proposed gene name	NCBI gene ID	Gene length (bp)	Exons	Putative protein length (aa)	*A. thaliana* / *A. lyrata* isotype	Chr, sub-genome
*CsTUG1-A*	104,699,727	3344	10	474	TUG1	7, G1
*CsTUG1-B*	104,749,225	3194	10	474	TUG1	16, G2
*CsTUG1-C*	104,788,465	3135	10	474	TUG1	5, G3
*CsTUG2-A*	104,708,550	2813	10	474	TUG2	8, G1
*CsTUG2-B*	104,734,567	2835	10	474	TUG2	13, G2
*CsTUG2-C*	104,768,758	2737	10	474	TUG2	20, G3

The exon-intron structures of the identified γ-tubulin genes of *C. sativa* were analyzed as well (Fig. S8). All six genes showed highly conserved exon-intron structure, consisting of 10 exons, despite their distinct isotype identity. The lengths of first and second introns varied significantly even within homeologous genes. A significant intron length variation was observed within intron3 of TUG2 (135–152 bp) and inton7 of TUG2 genes (107 bp in C subgenome v. 149 bp in A and B). Other introns of TUG1 and TUG2 genes varied only by few nucleotides in length (1–4 bp) or not varied at all.

The conserved motifs and domains of the identified γ-tubulins are shown in Fig. S9. Each of the identified γ-tubulin was found to contain 13 conserved motifs (Fig. S9a), as well as three typical domains: GTPase, Tubulin C-terminal and C-terminal tail domains (Fig. S9b and Fig. S9c). However, TUG1 and TUG2 isotypes can be effectively differentiated by their amino acid sequences. The distinction between these two isotypes was conditioned by only three amino acid substitutions of with different physic-chemical properties at the positions: 94 (TUG1 – A, TUG2 – S), 211 (G – N) and 455 (E – G), which was observed in *A. thaliana*, *A. lyrata* and *C. sativa*. Thus, 93 V in TUG1 appeared to be 93 L in TUG2 in the mentioned species, as well as at 392 K in TUG1 was substituted by R in *TUG2*. The position 81 of TUG1 and TUG2 was far less conserved among Camelineae and differed within the species. Only few positions of *CsTUG1-A/B/C* and *CsTUG2-A/B/C* contained specific amino acid residues distinct from those in the proteins of *A. thaliana* and *A. lyrata* (303, 432, and 464).

**Fig. 3:**
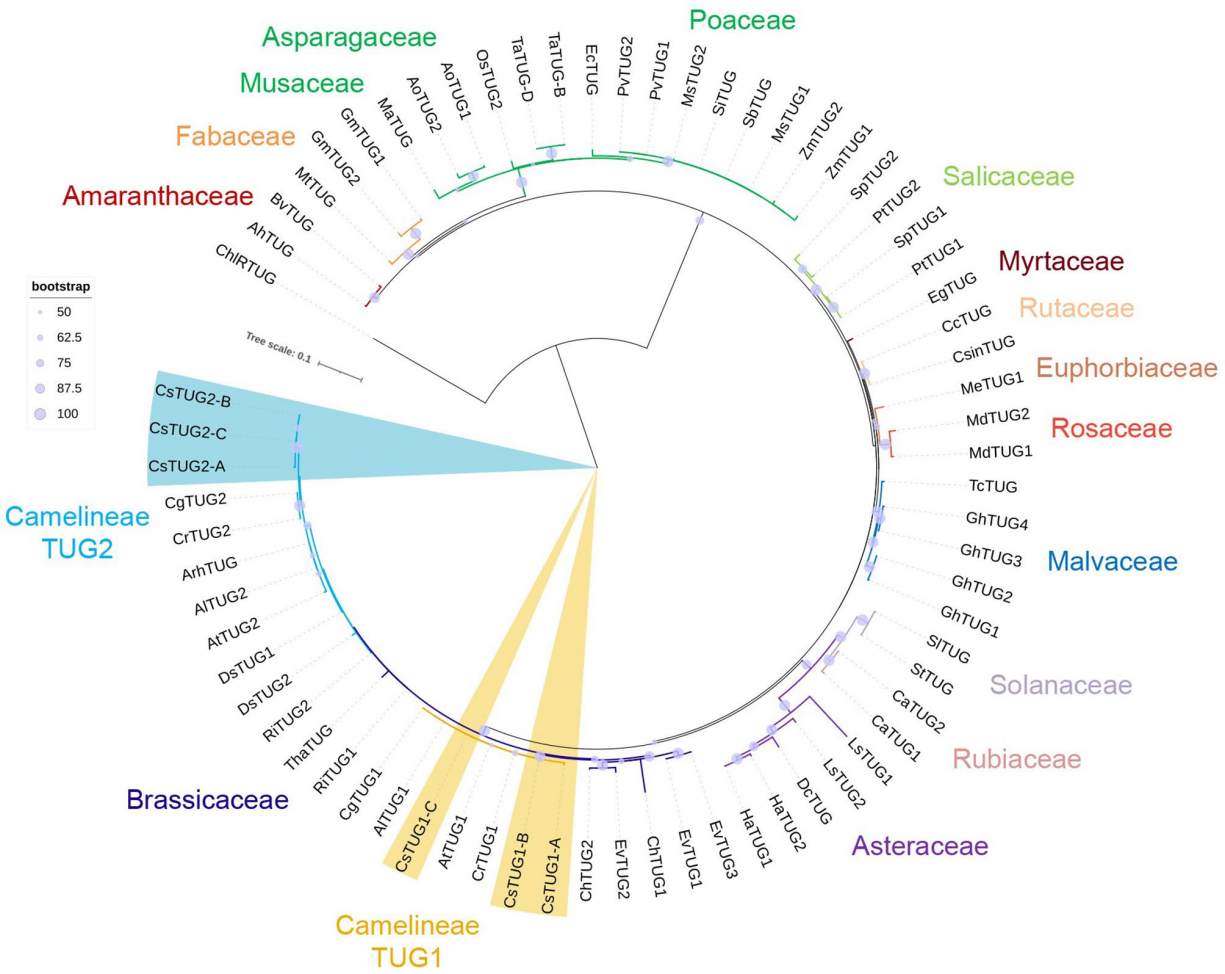
Phylogenetic analysis of plant γ-tubulins. Maximum likelihood analysis was performed involving 73 proteins sequences from 42 species, the gene list is provided in Table S6. The tree was constructed with the bootstrap support of 1000 iterations, low bootstrap values are excluded from tree representations

A total of 72 γ-tubulin protein sequences from 41 flowering plant species were used for phylogenetic analysis (Fig. 3), and the γ-tubulin sequence from *C. reinhardtii* was used as the outgroup (Table S6). Despite the genome of *C. sativa* has 6 different γ-tubulin proteins, the majority of flowering plant genomes typically contain 1 to 3 distinct γ-tubulin genes. Existence of two distinct isotypes (TUG1 and TUG2) in the genome of *A. thaliana* represents rather an exception, than the general rule for flowering plants, dicots, or even Brassicaceae family.

Results of γ-tubulin phylogeny reconstruction indicated that the orthologous groups TUG1 and TUG2 isotypes are limited only a particular group Brassicaceae, most likely within Camelineae tribe or other closely related species (Fig. 3). Clear separation into two distinct clades representing TUG1 and TUG2 isotype groups was observed only for γ-tubulins of *C. sativa*, *A. thaliana*, *A. lyrata*, *Capsella grandiflora*, *C. rubella* species. Most likely, these genes could result from an ancient WGD event and were later preserved in the genomes of the mentioned species. During our brief search for annotated γ-tubulins, only one TUG gene, *ArhTUG*, was identified for *A. halleri*. The *ArhTUG* peptide was placed together with TUG2 proteins of Camelineae (Fig. 3). Nevertheless, this question would require a separate comparative genomics analysis of *A. halleri* with its relatives.

No orthologous relationship was identified between TUG1, TUG2 proteins of *Rorripa islandica* and *D. sophoides* and the members of Camelineae TUG1 and TUG2 isotypes. Despite both *R. islandica* and *D. sophoides* species are members of Brassicaceae Lineage I, their TUG proteins were grouped separately from Camelineae TUG1 and TUG2 isotypes (Fig. 3). This can indicate that duplication of γ-tubulin genes in *R. islandica* and *D. sophoides* was driven by a potentially separate duplication event. The history of γ-tubulin genes multiplication in Brassicaceae (including representatives of other Lineages) is not completely clear and, again, might be investigated separately. However, it can be concluded that TUG1 and TUG2 isotype groups, identified for *A. thaliana* and other Camelineae, are not transient for other species, even within Brassicaceae family. No common isotype/class-specific grouping of γ-tubulins was observed for other plant families (Fig. 3), as it was in the cases of α- and β- tubulins (Figs. 1 and 2). In all other cases, TUG proteins were predominantly grouped in accordance with the taxonomy of the species, with some exceptions.

### Allocation of tubulin genes on ACK genomic blocks within chromosomes of C. sativa and synteny analysis

**Fig. 4:**
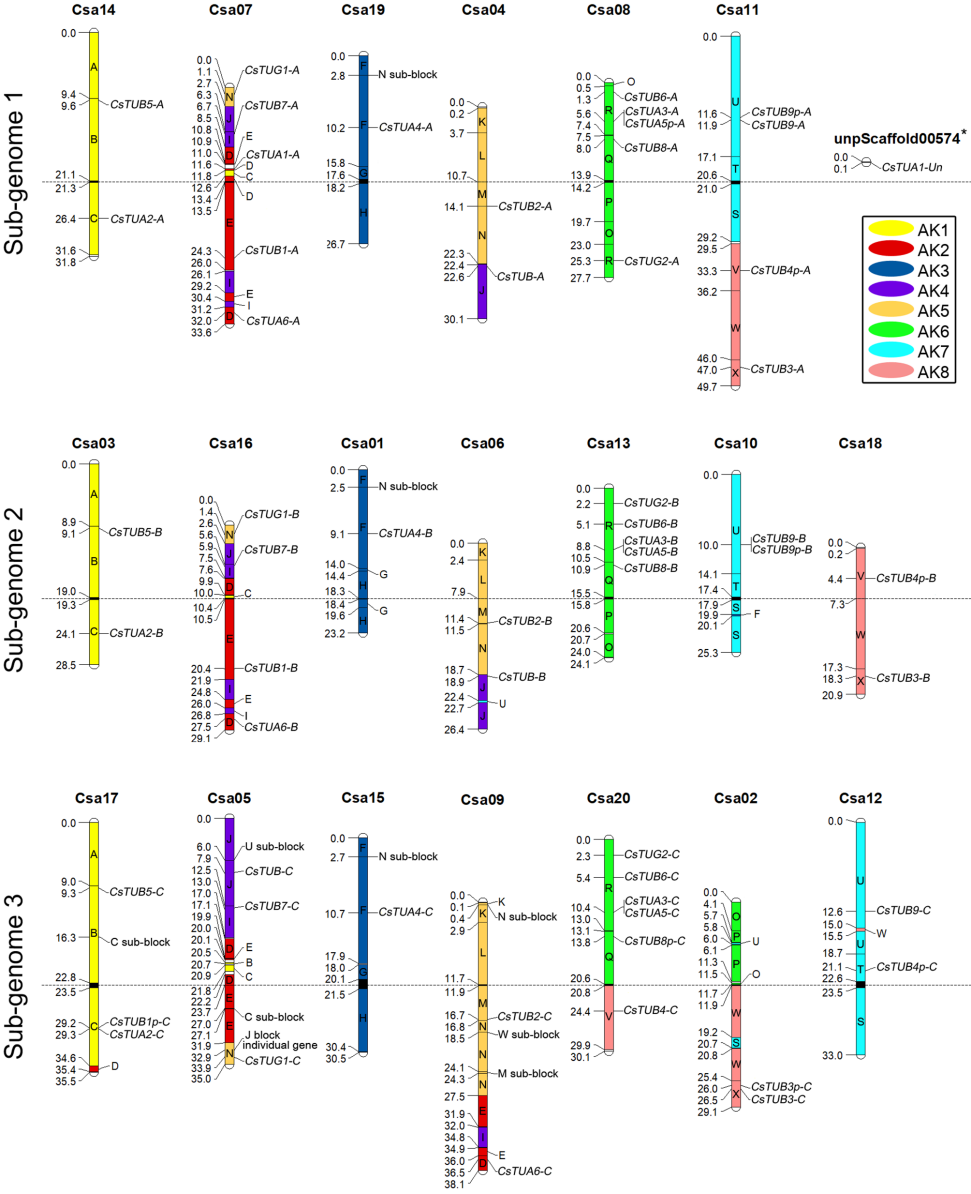
Ancestral crucifer karyotype (ACK) genomic blocks, mapped on *C. sativa* chromosomes with allocated tubulin genes. AK1-8 colors are referring to ancestral chromosomes, to which A-X blocks correspond. Centromeric regions are colored in black, while white indicates the regions that were not assigned to specific ACK block

The allocation of ACK genomic blocks on *C. sativa* chromosomes was reconstructed based on the available data on *C. sativa-A. thalina* syntenic loci pairs, assigned to a particular ACK block. This allowed identifying to which ACK genomic block a particular tubulin gene corresponds in the genome of *C. sativa* (Fig. 4). The reconstructed chromosome map with ACK genomic blocks has higher resolution and shows presence of several minor genomic block fragments.

The assignment of tubulin genes to ACK blocks allowed to confirm homolog of the majority of the gene duplicates, since they were usually contained in homologous chromosomal regions in distinct subgenomes. The triplets of tubulin homeologs, present in *C. sativa*, are listed in Table S7, as well as their orthologs from *A. thaliana* and *A. lyrata* genomes, which were identified via additional reconstruction of tubulin genes allocation in ACK blocks in *Arabidopsis* sp. genomes (Fig. S10).

Inferring the orthologs of tubulin genes on the macro syntenic scale confirmed the described above findings, which were based on genomic landscape differences among the genes encoding distinct tubulin isotypes. For example, the ortholog of *CsTUA2-A*/*B*/*C*, *AtTUA2* and *AlTUA2* was additionally confirmed, since all these genes were located in the homologous block C regions in different genomes. Similarly, orthologous *CsTUA4-A*/*B*/*C* and *AlTUA4p* are contained in block F of *C. sativa* and *A. lyrata* genomes. At the same time, such gene is absent in the F block of *A. thaliana*. The gene currently named *AtTUA4* is located in the block A, which may indicate its paralogous nature (possible duplicate of a TUA Class I gene). It was also confirmed that all genes of TUA6 isotype are contained in different genomic blocks of three mentioned species. Clarification of homology status of *AtTUA4* and TUA6 genes will require additional investigation, involving other *Arabidopsis* species.

The performed reconstruction of tubulin genes positioning within genomic blocks also clarified the nature of tubulin pseudogenes. For instance, while the majority of *CsTUB4* (pseudo)genes are contained in V blocks in different subgenomes, *CsTUB4p-C* is located in T block of the third subgenome, which suggests its paralogous status (Fig. 4). Another β-tubulin pseudogene from the third subgenome, *CsTUB1p-C*, seems to be lost in its original locus at the block E and “migrated” to the block C. Most likely, this pseudogene arose as the result of single gene duplication or partial translocation, therefore can be classified as the paralog for other *CsTUB1* representatives.

**Fig. 5:**
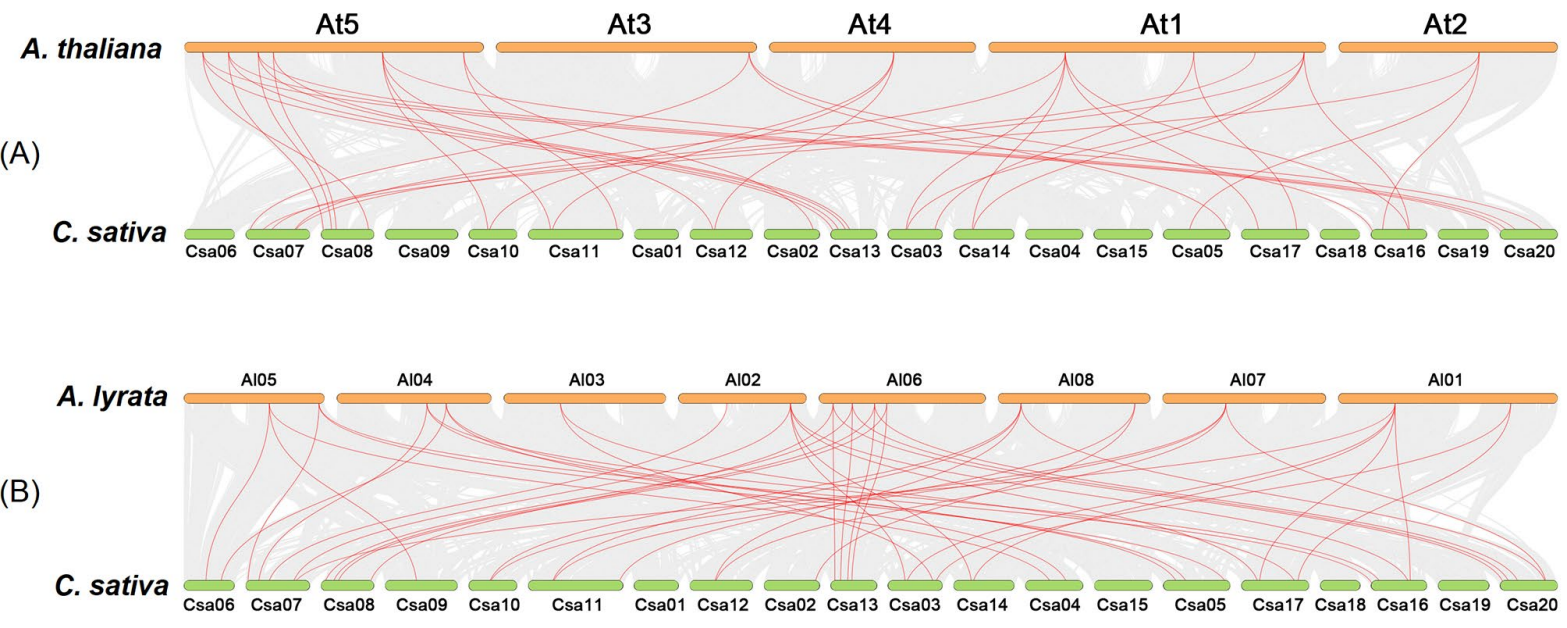
Synteny analysis of tubulin genes between *C. sativa* and two *Arabidopsis* species: **(a)** with *A. thaliana*; **(b)** with *A. lyrata*. The collinear blocks between *C. sativa* and *Arabidopsis* species are showed with gray lines, while syntenic pairs of tubulin genes were highlighted with red. The chromosome number is indicated above each respective chromosome

Despite the described peculiarities of orthologous relations between tubulin genes of *Camelina* and *Arabidopsis*, the whole-genome microsynteny analysis (Fig. 5) showed non-differentiated detection of syntelogs, which usually include both paralogs and orthologs. In some cases, members of one tubulin Class were defined as the syntelogs. For instance, the genes *AtTUB1*/*AlTUB1* and *CsTUB5-B* formed syntenic pairs. This serves as the additional evidence for the hypothesis that tubulin gene diversity within Classes originated in result of ancient duplication event. However, more often only genes, encoding a particular isotype, formed syntenic pairs. For instance, *CsTUB2-A/B/C* formed syntenic pairs, only with *AlTUB2*, but not with *AtTUB2* and *AtTUB3* (*AtTUB3-1*, *AtTUB3-2*) (Fig. 5), which is consistent with our previous conclusion made via the analysis of genomic context of these genes (Supplementary Note 2). Similarly, no α-tubulin genes of *C. sativa* formed any syntenic pair with *AtTUA4* and *AtTUA6*, both of which are contained in loci with distinct genomic context (Supplementary Note 1).

**Fig. 6:**
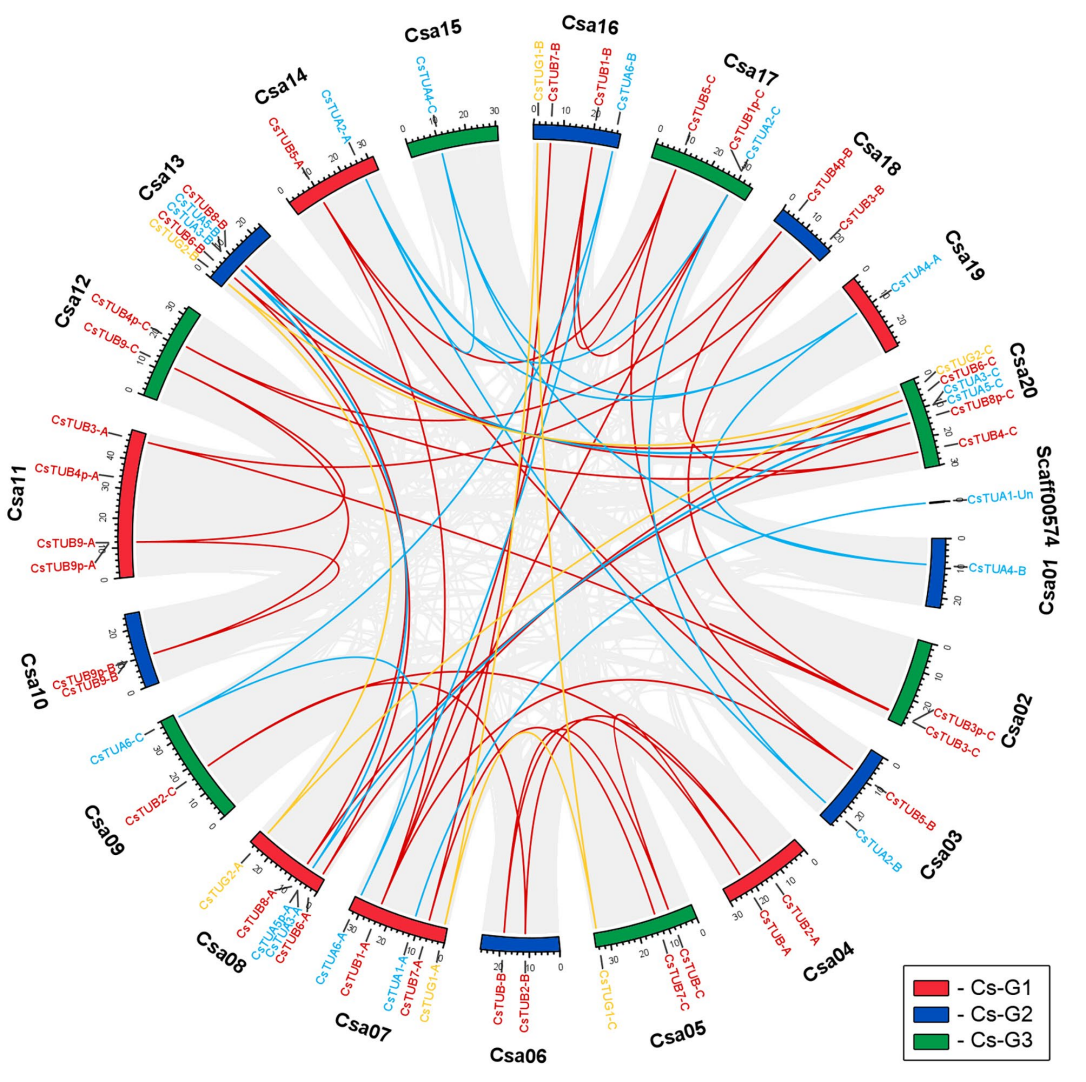
Synteny analysis of interchromosomal relationships of tubulin genes from different subgenome of *C. sativa*. All gene pairs were colored with gray, while α-tubulin genes with blue, β-tubulin – red, γ-tubulin – yellow. Chromosomes, belonging to different subgenomes were distinctly stained with, according to the three-color code, showed on the figure

Further, interchromosomal synteny of *C. sativa* tubulins was inferred (Fig. 6). In total, 5 α-tubulin homeologous triplets, 9 triplets of β-tubulin homeologs and 2 triplets of γ-tubulin genes were detected. In addition, three homeologous pairs were confirmed, accounting *CsTUA1-A* and *CsTUA1-Un* pair, homeoparalogous pair of *CsTUB9p-A* and *CsTUB9p-C* pseudogenes and a pair of *CsTUB1-A* and *CsTUB1-B* genes. Infragenomic synteny was rather precise and, in the vast majority of the cases, the identified syntelogs were homeologous genes.

The performed analysis also allowed clarifying the nature of some pseudogenes. For instance, *CsTUB4p-C*, located in atypical genomic block for TUB4 genes, formed syntenic pairs with the functional gene from the third subgenome, *CsTUB4-C*, and with the pseudogene from the second subgenome, *CsTUB4p-B.* Moreover, according to the results of genomic blocks reconstruction and synteny analysis *CsTUB3p-C* was found to be a tandem duplicate of *CsTUB3-C*, located on Chr02. The block X of Chr02 does not significantly differ in size, compared to homologous blocks in other subgenomes (3.6–3.7 Mbp), which excludes the possibility of a large scale duplication of this genomic region. Additionally, both *CsTUB3-C* and *CsTUB3p-C* are located closely (0.488 Mbp) to suppose that the later pseudogene arose as a result of tandem duplication at this chromosomal region.

Based on the inferred type of homology relations of the identified *C. sativa* tubulins among them and with the members of tubulin gene family in *A. thaliana* and *A. lyrata* (Table S7), we established the values of *Ka* and *Ks* coefficients (Fig. S11 and S12). Commonly, the rates of non-synonymous substitutions were very low (*Ka* < 0.04), close to zero among all tubulin subfamilies in *C. sativa*. At the same time, the present functional genes accumulated enough synonymous substitutions in the coding regions (*Ks* < 0.2) (Fig. S11). No significantly different Class- or isotype-specific sequence conservancy was detected for all tubulin subfamiliy, except the α-tubulins, which showed the *Ks* values for Class II homeologs (Fig. S11a). The pair of *CsTUA1-A/Un* genes showed the highest sequence divergence rate among all functional tubulin genes (*Ka/Ks* = 0.538). It is likely that the currently present functional tubulin genes in *C. sativa* are to be preserved (Fig. S11d). Interspecific comparison of tubulin gene orthologs showed the same pattern, where all tubulin genes tend to accumulate more synonymous substitutions (*Ks* < 0.5), rather than non-synonymous (*Ka* < 0.04) (Fig. S12). It is also noteworthy that γ-tubulin genes were the most conserved tubulin genes in the both comparisons (Fig. S11c, S11d and S12), while α- and β- tubulins show diverse rate of synonymous substitutions (Fig. S11a, S11b and S12).

### Expression profiling of tubulin genes based on transcriptomic data

**Fig. 7:**
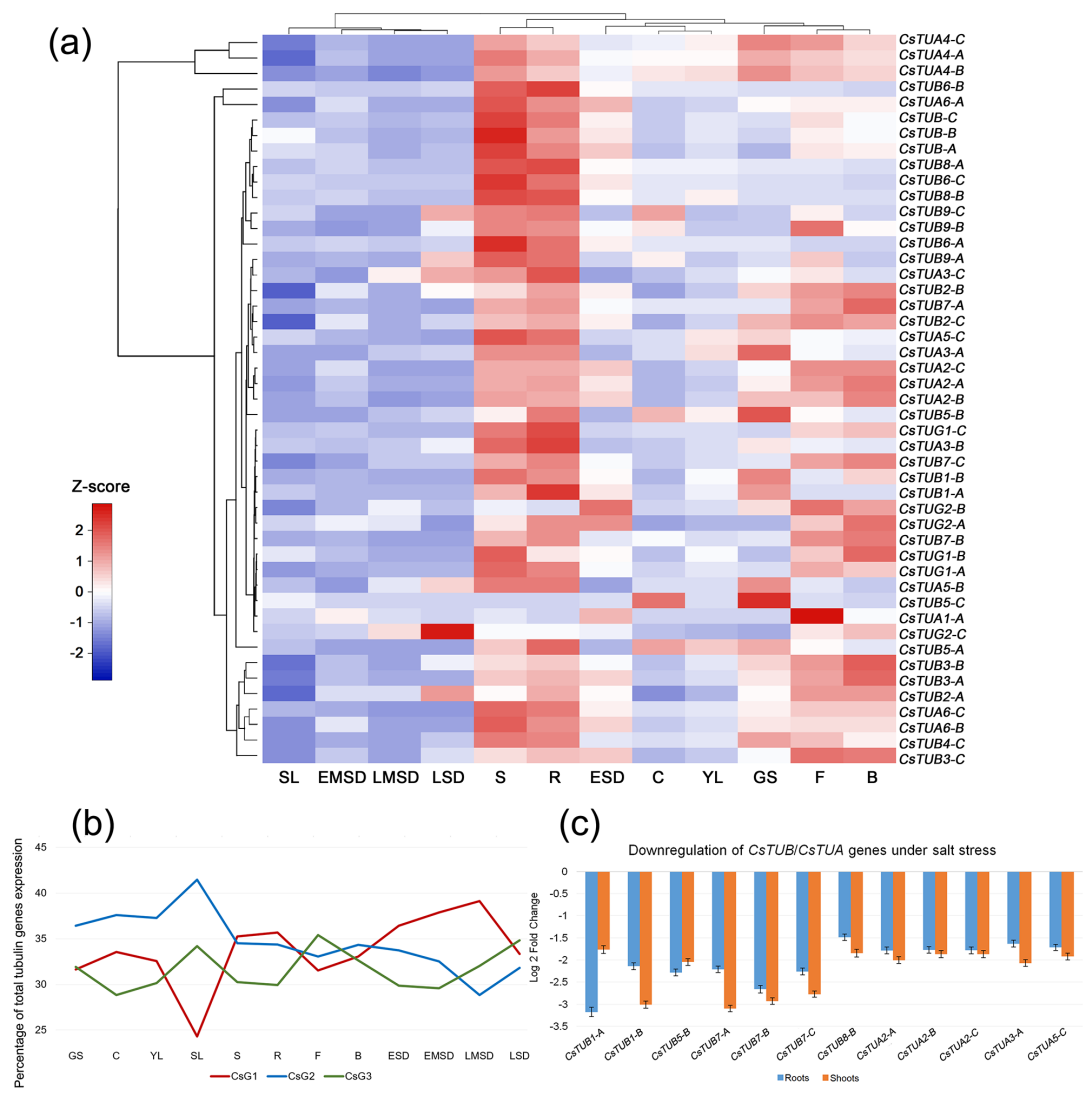
Expression of all identified *C. sativa* tubulin genes. **(a)** Heatmap, representing expression patterns of the identified functional tubulin genes in different tissues and on different developmental stages; **(b)** Diagram of contribution of each *C. sativa* subgenomes into the total expression of tubulin genes encoding functional proteins (*CsTUA1* homeologs were not included in this analysis, due to unclarified origin of *CsTUA1-Un*); **(c)** The most significant changes in tubulin genes expression under salt stress

We have investigated the expression of the identified tubulin genes in various tissues (Fig. 7). The majority of tubulin genes were characterized by the highest expression levels in stem and roots of mature plants, while the lowest transcript level was observed in senescing leaves and in maturing seeds during EMSD and LMSD. On contrary, during LSD the expression of numerous tubulin genes was upregulated, including *CsTUG2-C*, which demonstrated the most significant upregulation among all γ-tubulin genes at this developmental stage. Genes of *CsTUG2-A*/*B*, contrarily, were upregulated during ESD stage. The second highest transcript levels of tubulin genes were detected in flower buds and flowers. *CsTUA1-A* gene was expressed at the flowering stage at least two-fold higher level than in any other plant organ/growth stage. At the same time, this tubulin gene was almost not transcribed in roots, stems, and germinating seeds, in which tubulin gene transcripts are quite abundant.

Triplets of *CsTUA2-A*/*B*/*C*, *CsTUA4-A*/*B*/*C*, and *CsTUB10-A*/*B*/*C* homeologs showed almost similar patterns of expression in all analyzed tissue types at various stages. The duplets of *CsTUB1-A*/*B*, *CsTUB3-A*/*B*, *CsTUB9-B*/*C*, *CsTUG1-A*/*B*, *CsTUG2-A*/*B* genes also followed the mentioned expression pattern of other tubulins. Paralogous *CsTUA3* and *CsTUA5* genes not always shared the expression patterns. However, homeo-paralogous *CsTUA3-A* and *CsTUA5-C* genes also were clustered together on heatmap (Fig. 7a). Despite that, highest absolute rates of β-tubulin transcript level were found in stem and root tissues and were held by the triplets of *CsTUB6* and *CsTUB10* homeologs, and by singleton *CsTUB4-C*. Among α-tubulins the absolute maximum of transcript levels was detected for *CsTUA4* and *CsTUA6* homeologs, also in stem and roots. It is also important to note that the mentioned α-tubulin genes were constantly expressed at the high levels in different tissues and likely are not specifically upregulated in stem or root.

The majority of tubulin pseudogenes are still expressed in *C. sativa*, except the *CsTUB9p-B*. All other pseudogenes produce detectable transcripts, while *CsTUB4p-A*/*B* pseudogenes were expressed on the comparable level with their functional homeolog, what may indicate that their pseudogenization had occurred relatively recently.

Since *C. sativa* has allohexaploid genome structure, we have investigated the contribution of each of three subgenomes into the total transcript level of tubulin genes (Fig. 7b). It was identified that the expression of tubulin homeologs is being unequal for different subgenomes. For the tubulin genes family, only slight domination of third subgenome was observed at flowering stage (35.4%) and during late seed development (34.8%). Despite that, in root and stem tissues the level of Cs-G3 tubulin transcript was significantly lower (29.9%) than that in other two subgenomes. During seed germination, as well as in cotyledons, young leaf and even in senescing leaf transcripts of second subgenome were the most abundant (36.4–41.5%). Transcripts of Cs-G1 tubulins were predominant from early to late-mid seed development (36.4–39.1%) with the increasing manner of first subgenome role with time.

As an example of tubulins expression changes under abiotic stress conditions, we have investigated the changes in *TUB*/*TUA* transcript levels under salt stress (Fig. 7c). It was found that *CsTUB1-A*/*B* and *CsTUB7-A*/*B*/*C* genes were synchronously downregulated (by 1.5- to 3-fold) both in roots and in shoots. Moreover, the mentioned genes were the most downregulated tubulins under the salt stress. In addition, some of the mentioned genes appeared to be differentially regulated in roots and shoots of young *C. sativa* plants. For instance, *CsTUB1-A* was significantly more downregulated in roots, while its homeolog, *CsTUB1-B*, showed more significantly decrease of the expression in shoots. This may serve as a very good example of tubulin homeolog subfunctionalization via the divergence of expression patterns. As for α-tubulins, a complete triplet of *CsTUA2* genes was downregulated in shoots and roots, while also *CsTUA3-A* and *CsTUA5-C* were both downregulated predominantly in shoots. None of the identified tubulin genes was found to be upregulated under the increased salinity conditions.

## Discussion

### The diversity of the identified tubulins

Successful identification and meticulous analysis of tubulin gene family in *C. sativa* allowed proper isotype designations of the identified genes. Moreover, clear orthologous relations of the identified genes with tubulins of *A. thaliana* were revealed. This is an important step towards understanding the functional roles of particular tubulin isotypes in flowering plant species. Commonly, identified plant tubulins are named in numerical order, but not in accordance with their orthologous relations, which appears to be a non-trivial task. More commonly, tubulins classification is limited to assigning genes to α, β and γ subfamilies and identification of major Classes within the subfamilies [7–9, 19, 24, 29, 32].

The lack of the information on tubulin orthologs is the main factor, limiting identification of particular isotypes. Moreover, the widely known model species possess not completely representative gene set of tubulin isotypes. In particular, it was shown that *AtTUA4*, *AtTUA6* do not have orthologs in the other species. At the same time, *A. thaliana* genome lacks gene orthologs of TUB2 and TUB (TUB10) isotypes. Previously, we have demonstrated that *AtTUA4*-*AtTUA6*, *AtTUA3*-*AtTUA5* and *AtTUB2*-*AtTUB3* are the pairs of relatively recent paralogs [80]. While *AtTUA3*-*AtTUA5* and *AtTUB2*-*AtTUB3* are local duplicates, the pair of *AtTUA4*-*AtTUA6* arose as the transposed duplicates of *AtTUA2*, most likely after the divergence of *A. thaliana*, since no orthologs for *AtTUA4* and *AtTUA6* were found neither in *A. lyrata* or *C. sativa* (in this study), nor in more distant species, like *Theobroma cacao* and *Vitis vinifera* [80]. These facts should be taken into account when extrapolating the results of cytoskeletal research to other species. Also, such orthologs of unobvious tubulin genes should be accounted if the set of tubulin genomes is used for the species phylogeny reconstruction.

An important feature of the tubulin family in *C. sativa* is high number of complete triplets of tubulin genes, accompanied by the high sequence conservancy. Despite the extremely conserved sequences, γ-tubulins are retained as two complete triplets per isotype. The encoded γ-tubulin peptides appeared to be almost identical within each triplet. The preservation of all six γ-tubulin genes may be associated with extremely important role [81], but it is still unclear, why all copies of the genes remained intact, since no significant divergence in their expression profiles were detected (Fig. 7a).

The majority of α-tubulins were also represented by complete triplets of functional genes in the genome of *C. sativa*. Only one pseudogene (*CsTUA5p-A*) from A subgenome was found. The remaining gene set of TUA3 and TUA5 isotypes remained unchanged. Most likely, *CsTUA3-A*/*B*/*C* and *CsTUA5-B*/*C* have diverged by their expression profiles, since they encode almost identical proteins in *C. sativa*. None of other TUA genes was pseudogenised or lost.

The case of *CsTUA1* remains unclear. *C. sativa* contains at least two genes of this isotype, one of which remains unplaced in the genome. Loss of potential third *CsTUA1* could be caused by several reasons, including its loss during numerous *C. sativa* chromosome shattering events [63, 64]. *CsTUA1-A* gene is located between D and E ancestral blocks (Chr07) near the ancient pericentromeric region of AK2 ancestral chromosome (Fig. 4). The orthologous *AtTUA1* and *AlTUA1* possess similar location (Fig. S10). Such locations of these genes, possibly, may have caused the loss of *CsTUA1* copies in B or C (sub)genomes during the degradation of the ancestral AK2 centromere. Also, lower gene density and high amount of tandem repeats may significantly complicate assembly of such regions, which could be the reason for the misassembly of the scaffold, containing *CsTUA1-Un* gene.

The subfamily of β-tubulin genes is characterized by the highest number of pseudogenes (Table S5), while the majority of pseudogenes is contained in C subgenome, which is believed to be the ‘youngest’ subgenome of *C. sativa* [37]. However, at least one member of each β-tubulin isotype remained in the genome, as well as the vast majority of isotypes (seven of ten) were represented by complete gene triplets (Table S7). Besides, *C. sativa* was found to contain an additional tubulin isotype, TUB10, if compared to *A. thaliana* genome (Table 3, S7). Another close relative, *A. lyrata*, and more distant species seem to have *TUB10* gene in at least one copy (Table S3). It is likely that the gene of TUB (TUB10) isotype was eliminated in the genome of *A. thaliana*, probably during divergence of this species, which was accompanied by loss of relatively large part of the genome (125 Mb of *A. thaliana* vs. 250 Mb of *A. lyrata*), involving coding sequences [82, 83]. Nevertheless, the loss of hypothetical *AtTUB* (*AtTUB-10*) seems to be not crucial for normal functioning of cytoskeleton or, probably, functional role of this isotype was compensated by other members of β-tubulin gene family.

Contrarily, *C. sativa* genome contains three homeologous copies, none of which was pseudogenised, contrarily to tubulin genes of other isotypes. Additionally, it is worth to mention that *AlTUB* and *CsTUB-A*/*B*/*C* proteins possess unique C-terminal tail sequence, which is different from other β-tubulin isotypes. Since C-terminal tail of β-tubulins is specific region, which serves as the target for various posttranslational modifications (PTM) [84], it may be possible that *CsTUB-A*/*B*/*C* proteins could have unique PTM and specific function, different from other β-tubulin isotypes.

### Expressional speciation of tubulin genes in allopolyploid *C. sativa* genome

The allohexaploid *C. sativa* species has inherited two subgenomes from *C. neglecta* and *C. neglecta*-like species (first and second subgenomes, A and B here, or N^6^ and N^7^) and one from *C. hispida* (the third subgenome, C or H^7^) [36, 37, 64]. Therefore, here we have defined all tubulin gene triplets as completely homeologous, but not ohnologous. The fact that the majority of the gene triplets retained without significant sequence divergence is interesting and suggests that presence of the same tubulin gene in multiple copies may provide a selective advantage. Non-elimination and sequence conservancy of tubulin gene duplicates (both homeologous or ohnologous) were observed in other polyploid plant species such, as cotton [24], flax [7, 10], *Populus* and *Salix* genera [9, 29] and *M. domestica* [32]. Moreover, it seems that the genes of particular isotypes are being conserved in polyploids, regardless of the time of genome scale-up event. However, this hypothesis requires further investigations on a broader species panel.

It is interesting to note that the most ancient, the second, subgenome of *C. sativa* shows the lowest rate of tubulins pseudogenization. At the same time, the third subgenome is characterized by the highest number of tubulin pseudogenes, despite this subgenome has been gained relatively recently [37]. Furthermore, the third subgenome of *C. sativa* had the lowest contribution to the total tubulins expression (Fig. 7b). Since the majority of the tubulin transcripts are coming from first and second subgenomes at curial developmental stages, the contribution of third subgenome appears to be not so important and, thus, pseudogenization of tubulin genes from C subgenome cannot affect dramatically of the vitality of *C. sativa* plants, carrying such disrupted genes.

Mutated or partially disordered tubulin proteins may cause the ‘spoiling’ of α/β dimers, decreasing strength of interdimer and/or intradimer contacts in microtubules [85], which may lead to lethal mutations. However, a mutation of a single copy of a particular isotype gene it may be not so critical for hexaploids, since there are still two more functional copies would be left, compensating the loss of homeolog/ohnolog. The preserved functional gene copies may majorly recover the formation of normal dimers. This could be one of the factors explaining, why some of tubulin gene triplets in *C. sativa* are mainly reduced to pairs, but not to the singleton genes of a particular isotype.

For instance, pairs of α-tubulin ohnologs in flax [7, 10] almost have not experienced pseudogenization, while only one β-tubulin isotype was represented by a singleton gene. Moreover, non-reduction of additional genes in α/β-tubulin gene family and incorporation of novel isotypes (via genetic engineering) into the microtubule assembly was previously not once described [3]. This could be partially explained by the expressional regulation of tubulin genes. It is believed that the tubulins expression is mainly guided for maintaining general balance between number of α and β monomers in the cell. Under this hypothesis, the possible expression of a specific isotype at particular developmental stages could be balanced by the changes of the transcript levels of tubulins from α and β subfamilies. The observed rapid expansion of tubulin genes family in flowering plants after the series of WGD events [86] is consistent with the tubulins transcriptional balancing theory, as it explains well the incorporation and non-reduction of tubulin gene duplicates.

In the vast majority of the cases *C. sativa* tubulins are expressed from *C. neglecta* and *C. neglecta*-like subgenomes (Cs-G1 and Cs-G2), thus it may be supposed that the results of microtubule-related investigations on this ancestor can be highly transferable to *C. sativa*. Such research becomes even more interesting in light of Cs-G1 tubulin expression dominance at almost all stages of seed development, when neutral lipids are actively deposited in lipid droplets, motility of which is highly related on microtubules functioning [87, 88]. This suggests that lipid droplets dynamics may be successfully studied not only on *C. sativa*, but also on its close diploid relatives, such as *C. neglecta*.

Despite the subgenome dominance in terms of the tubulins expression, the majority of homeologous tubulin triplets were synchronously downregulated under the increased salinity (Fig. 7c). A recent paper suggests that salt tolerance in *A. thaliana* is associated with increased expression of TUB3, TUB4, TUB7 and TUB9 isotypes in vitro [89]. In addition, it is worth noting that TUB7-deficient *(A) thaliana* mutant have shown normal phenotype under increased salinity, while loss of function of TUB9 (which is not been found to be downregulated in *C. sativa*) resulted in hypersensitive phenotype, while its overexpression rescues the phenotype. Homolog (possibly ortholog) of TUB7 isotype was also found to be downregulated in the *(B) napus* [90]. Thus, it can be assumed that TUB7 could be one of the most important β-tubulin isotypes that are involved in microtubule functioning under the salt stress, while the in vitro upregulation of *AtTUB7* can be not completely representative for the in vivo system.

While such homeologs as *CsTUB7* were synchronously downregulated, there were several individual genes of other TUB isotypes, which showed decrease in the expression under the salt stress (Fig. 7c). Among them *CsTUB5-B* and *CsTUB8-B*, orthologs of which were not previously found to show the altered expression in *A. thaliana* under the increased salinity [89]. Previous results suggested that in *A. thaliana* the *AtTUA6* gene was upregulated under the salt stress [91]. However, as it mentioned above, this gene does not have any orthologs in even closely related species. Furthermore, we have not found any upregulated tubulin gene under the increased salinity, suggesting that the mentioned upregulation of *AtTUA6* may be its specific feature. In the same research the downregulation of TUB2 was also detected [91], which was not observed for any CsTUB2 homeologs in *C. sativa* in present study.

### Tubulin phylogeny and possibility to exploit tubulin polymorphism

Phylogenetic analyses of α-, β- and γ-tubulins raised questions about the origin of the tubulin isotype diversity not only in *C. sativa*, but also in various flowering plant groups. It was found that α-tubulins are represented by two distinct phylogenetic Classes (Fig. 1). The α-tubulin genes that belong to distinct phylogenetic groups show clearly different exon-intron structures, which is being conserved among the representatives of a particular Class (Fig. S3). Such findings are consistent with the previous results, obtained for various plant species [19, 29]. However, the reason for such differentiation remains unclear.

Similarly, the exon-intron structures of β-tubulin genes tend to be extremely conserved (with few exceptions) [3], the understanding of the role of such a high isotype diversity is still very limited. The members of sub-Class I-like are typically associated with secondary cell wall development [29]. This phylogenetic group could represent specialized Class I proteins that could be possibly involved in the determination of the orientation of cellulose microfibrils in plant secondary fiber cell walls [92]. Representatives of Class I-like were found in woody species, like *Populus* [9] and *Salix* [29], as well as proteins of these sub-class were also found in species, which are developing strong fibers, like flax [7, 10] and cotton [7, 9, 19, 24, 29]. It can be concluded that the role of Class I-like β-tubulins has not been enough clarified yet and may be the subject of further investigations.

Additionally, the role of TUB10 isotype remains unclear, as its loss in *A. thaliana* genome appears to be not crucial for the plant survival. The potentially preferential conservancy of certain isotypes appears to be also questionable (Table S7). Previously, it was reported that tubulin genes might have tissue specificity in *A. thaliana* [19]. Such genes as *AtTUA1* are preferentially expressed in pollen, which was also observed in the present study for its ortholog, *CsTUA1-A* (Fig. 7a). High tissue-specificity of the tubulin gene expression most likely play an important functional role (e.g. pollen maturation, etc.). In *A. thaliana*, the *AtTUB9* (Class III) serves as the role of pollen-specific β-tubulin [19], while in the present study only *CsTUB9-B* was somehow higher expressed during in flower tissues (Fig. 7a). However, the majority of the identified genes were not exclusively expressed in particular tissues. It is also worth noting that in rice *OsTUB8* (Class IV) was found to be predominantly expressed in anther (with mature pollen) and were hardly detectable in other tissue [28]. Albeit, no definitive conclusion could be drawn even for pollen specificity of certain β-tubulin isotype lineage or Class, since the described *AtTUB9* and *OsTUB8* belong to distinct phylogenetic groups (Classes III and IV, respectively) (Fig. 2). Unfortunately, there is still poorly understood about the functional role of particular isotype, as the many cytoskeleton studies are mainly focused on the whole microtubules themselves.

Higher number of β-tubulin genes in various plant taxa, extremely conserved genomic organization of the genes and high intron length variability conditioned the usage of β-tubulin intron regions as molecular markers [3, 79, 93]. Previously, conserved exon position allowed partial sequencing of 20 β-tubulin genes of *C. sativa* and several gene sequences from other *Camelina* species [50]. Later, it was shown that the β-tubulin based marker system is capable of differentiating *Camelina* species, especially taxa with different ploidy, as well as different cytotypes of the same species [94]. Moreover, we demonstrated possibility to use such markers for *C. sativa* cultivars genotyping [95] and for genetic polymorphism assessment of wild *Camelina microcarpa* germplasm [96]. Such approach allows assessing β-tubulin intron polymorphism simultaneously at multiple loci, which are located in different genome regions, on different chromosomes (Fig. 4). Such combined analysis is more informative than other molecular marker systems (e.g. SSR), as well as this method appears to be cheaper, faster and more convenient for dealing with the samples with shattered DNA, e.g. herbarium specimens [93, 96, 97].

Finally, phylogenetic analysis of plant γ-tubulins revealed that the presence of two distinct isotypes appears to be typical only to a limited number of Brassicaceae species (some Camelineae and several taxa of Brassicaceae Lineage I) (Fig. 3). However, most other flowering plants have not shown the presence of any isotype Classes. Multiple copies of γ-tubulins observed in *T. aestivum*, *G. hirsutum*, *M. sinensis*, *Salix purpurea*, etc. are most likely resulting from WGD or other gene duplication events, rather than from orthologous inheritance of distinct isotypes. It is unclear, why *A. thaliana* (and its relatives) has two distinct isotypes that are both conserved [81, 98], while, for example, in flax the second copy of γ-tubulin arising from WGD was disrupted and pseudogenised [7]. Moreover, it was shown that both *AtTUG1* and *AtTUG2* isotypes are needed for normal plant development [81], as well as for adequate stress responses, like the development of functional syncytium, induced by parasites [99].

The topology of the reconstructed phylogenetic tree of γ-tubulin mostly represented taxa-specific group of flowering plants. Potentially, γ-tubulins could be used for plant phylogeny reconstruction, due to high sequence conservancy of these genes (Fig. S12). Enough clear orthology of γ-tubulins, slow sequence evolution and limited variation of gene copies number (one per genome/subgenome) (Fig. 3) makes them attractive candidates for such task. In addition, β-tubulins were also successfully used for phylogeny reconstruction of *Ceratocystis* species [100]. However, the usage of β-tubulin in phylogenomics appears to be more complicated, since it requires proper understanding of β-tubulin isotypes orthology in analyzed species, which is hard to establish without having reference genome. On contrary, γ-tubulins appear to be more suitable for phylogeny due to more obvious orthology among different plant groups.

## Conclusions

In the present study, we report about complete identification and characterization of tubulin gene family in *C. sativa*. The exon-intron organization, phylogeny and synteny of the identified genes was analysed as well. In total, 17 α-, 34 β- and 6 γ-tubulin genes were identified, all of which were assigned to a particular isotype (using *Arabidopsis thaliana* and *A. lyrata* as the references), based on the tubulin gene orthology. The applied technique allowed not only identifying *C. sativa* tubulin orthologs in model *Arabidopsis* species and tracking tubulin gene evolution, but also uncovered that *A. thaliana* is missing orthologs for two β-tubulin isotypes, while two its α-tubulin genes are of ancient paralogous nature and do not have orthologs in other investigated Brassicaceae species. The reported results deepen our understanding of tubulin evolution, which are the key component of microtubules, a crucial part of cytoskeletal system in eukaryotes. Furthermore, the inferred orthology of tubulins opens possibility for understanding the functional roles of particular tubulin isotypes. Apart from that, characterization of tubulin gene family in *C. sativa* allows their usage as precise molecular markers for species barcoding and genetic diversity assessment, as it was previously shown for many flowering plant taxa.

### Electronic supplementary material

Below is the link to the electronic supplementary material.

Supplementary Material 1

## Data Availability

Whole genome sequence information for *C. sativa* (cv. DH55) (GCA_000633955.1) was obtained from the NCBI Genome database (https://www.ncbi.nlm.nih.gov/datasets/genome/) and EnsemblPlants database (http://plants.ensembl.org). All accession numbers of the sequences, used for phylogeny reconstruction are listed within Table S1-S4 and Table S6. The transcriptomics data of *C. sativa* (cv. DH55) were obtained from BAR ePlant database (https://bar.utoronto.ca/) and supplemental dataset, published by Heydarian et al. (https://doi.org/10.1038/s41598-018-28204-4). The datasets supporting the conclusions of this study are included in the article and in additional files.

## Abbreviations

TUA: α–tubulin
TUB: β–tubulin
TUG: γ–tubulin
WGD: Whole genome duplication
