# Supplementary material for: Genome-wide identification and evolution of the tubulin gene family in Camelina sativa
Source: BMC Genomics. 2024 Jun 14;25:599. doi: 10.1186/s12864-024-10503-y (PMC11177405; doi:10.1186/s12864-024-10503-y)
Supplement: Supplementary file 1 — Supplementary Material 1 [file 12864_2024_10503_MOESM1_ESM.docx]

**Supplementary file 1**

**Identification, characterization and isotype classification of the identified α-tubulins**


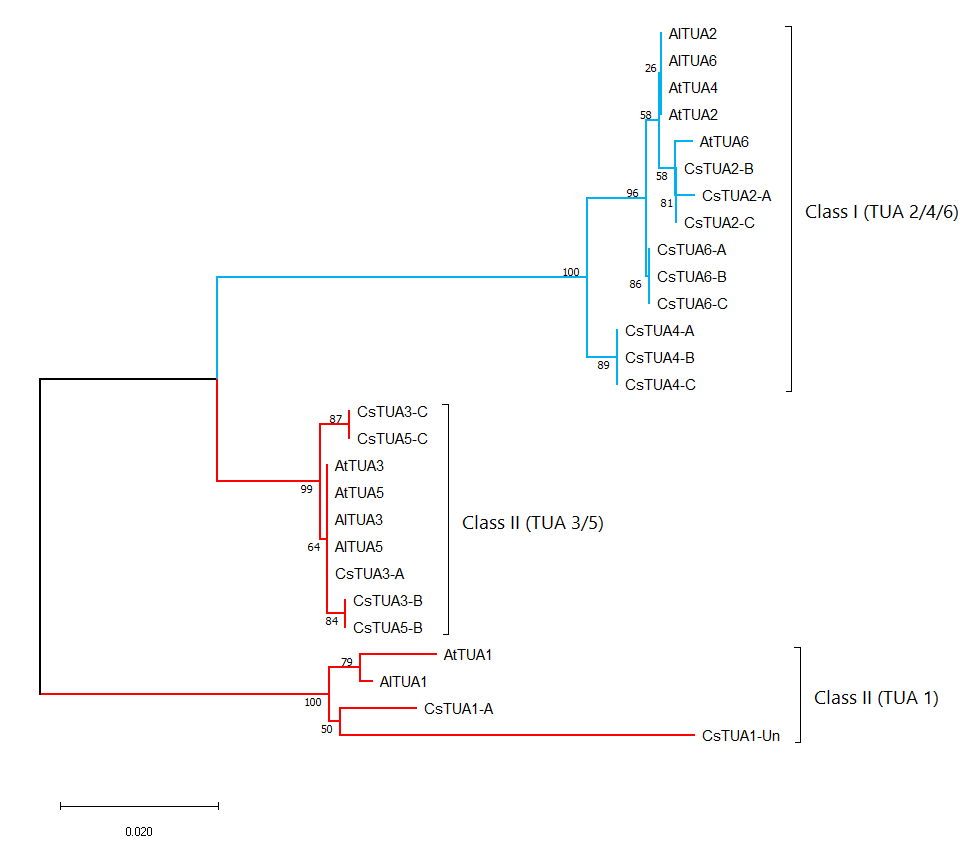


**Figure S1.** Phylogenetic tree (NJ) of *A. thaliana*, *A. lyrata* and *C. sativa* TUA protein sequences with 1000 replicates bootstrap support. Initial identification of TUA isotypes was conducted, basing on the results of this analysis.


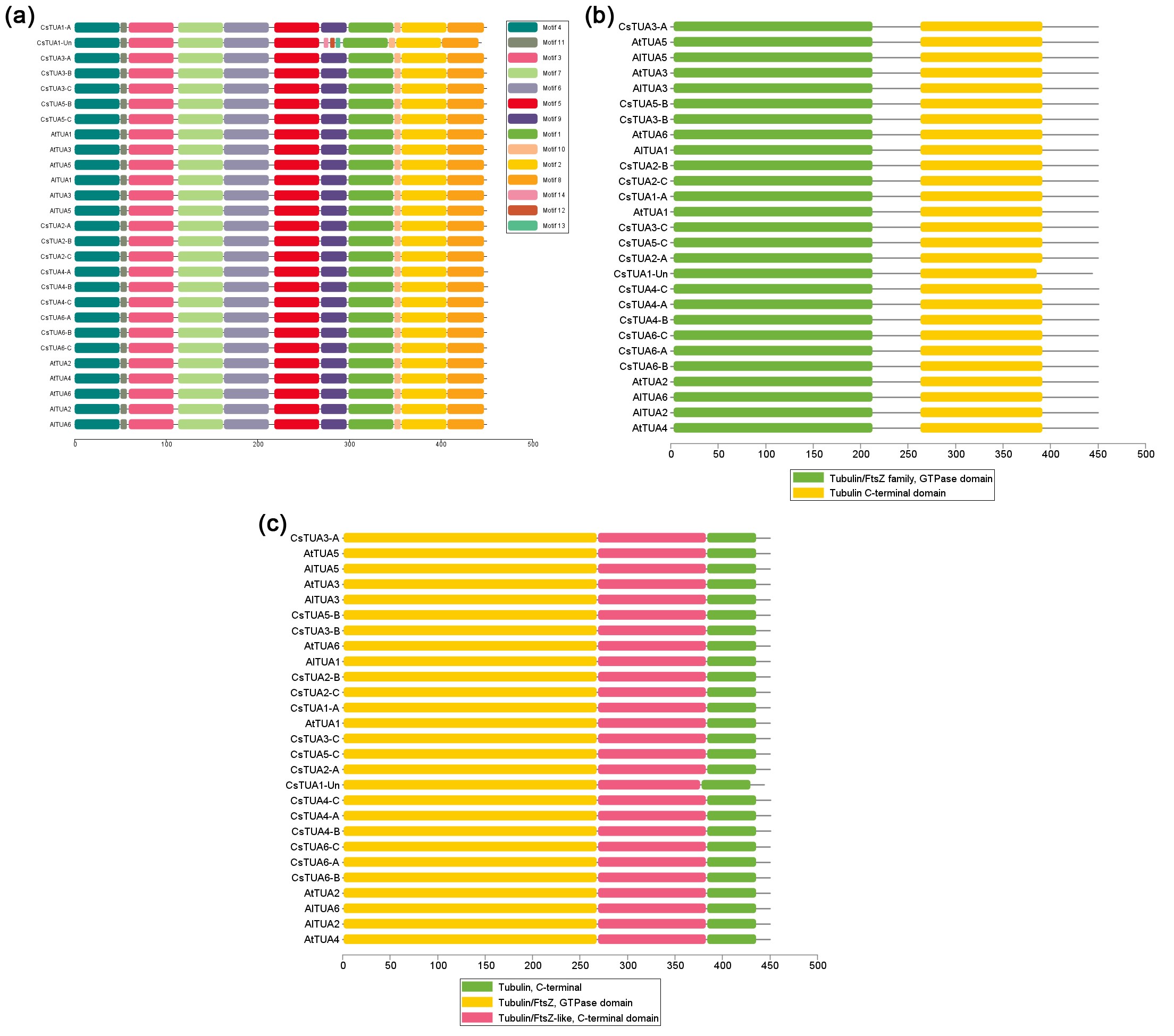


**Figure S2.** Distribution of conserved sequence motifs and functional domains within the identified α-tubulin peptides. **(a)** Conserved peptide sequence motifs in the identified α-tubulins; **(b)** functional domains of the α-tubulins identified against Pfam database; **(c)** structural domains identified against CATH-Gene3D database.


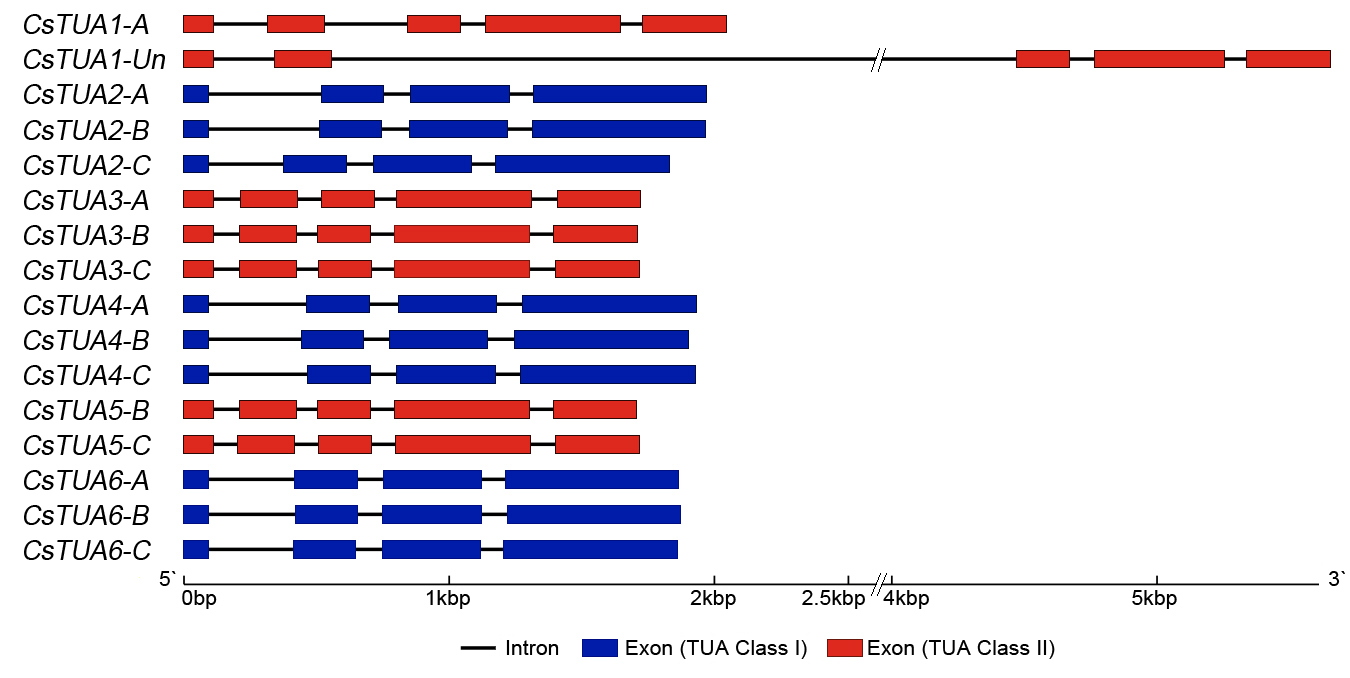


**Figure S3.** Exon-intron structure of identified α-tubulin genes in the genome of *C. sativa*.

**Supplementary Note 1.** Loci differences within the α-tubulin genes of *C. sativa* and related Brassicaceae species

**Locus of TUA3 and TUA5 palaeoparalogs in *C. sativa*, *A. thaliana* and *A. lyrata***

As an alternative approach, we have investigated the differences between the genomics landscape at the loci of potentially orthologous genes of various tubulins isotypes in the genomes of *C. sativa* and *Arabidopsis* species.

For example, unlike *TUA3*, the 3`-end of *TUA5* genes is always located near the locus of *RAP2-11* (AT5G19790, ethylene-responsive transcription factor RAP2-11). Tendency for the co-localization of these genes is observed in both *A. thaliana* and *A. lyrata* genomes (*RAP2-11* NCBI gene ID: 832099 and 9307976, respectively. Based on this fact, *CsTUA5* genes were distinguished from *CsTUA3* genes in the genome of *C. sativa*, as well as *AlTUA5* (9310031) in the genome of *A. lyrata*. It is important to note that *CsTUA3* and *CsTUA5* genes are encoding very similar proteins (99.56-100%), which usually leads to misidentification of these genes during genome annotation of different species. Such close allocation of *AtTUA3* and *AtTUA5* in the genome (and their orthologs in other species) strongly suggests paralogous nature of these genes. Nevertheless, it is important to distinguish these genes. It seems that the relations between *AtTUA3* – *AlTUA3* – *CsTUA3-A*/*B*/*C* are being strictly orthologous, which it crucial to consider, in order to properly extrapolate results, obtained on model species.

Other identified α-tubulin in the genome of *C. sativa* belongs to TUA1 isotype (from Class II), which seems to have diverse genomic context, but usually it is a glucan endo-1,3-beta-glucosidase 8 (homolog of AT1G64760) gene on 3`-side of *TUA1* gene in *A. thaliana* (842784) and in *A. lyrata* (9324342). In *C. sativa* genome, endo-1,3-beta-glucosidase 8 gene (104700953) is located from 5`-side of *CsTUA1-A*, what suggest that *TUA1* gene is inverted in A-subgenome of *C. sativa*. For example, another gene, *ILITHYIA* (AT1G64790, 842787) is located distantly from 3`-side of *TUA1* in the genome of *A. thaliana* and in *A. lyrata* (ILITHYIA gene - 9324350). At the same time, this gene is also located on 5`-side from *TUA1* of *C. sativa*, what also marks the inversion only of *CsTUA1-A* at this locus. It is hard to compare peculiarities of TUA1 location in B and C genomes of *C. sativa*, since, except *CsTUA1-A*, only one more gene was identified - *CsTUA1-Un*, which is located in unplaced scaffold. Due to this reason, it cannot be revealed, to which subgenome *CsTUA1-Un* gene does correspond, as well it is hard to clarify its genomic context. Possible loci for other two homeologs of *CsTUA1-A* will be discussed later below.

**Loci of α-tubulin Class I members in *C. sativa* and related Brassicaceae species**

Differentiation of isotypes of TUA Class I was less obvious than Class II, as it was previously demonstrated that TUA Class I proteins and their genes are even more conserved than Class II representatives, which may lead to inaccurate annotation of these genes. *A. thaliana* has three TUA Class I genes and one pseudogene of TUA2, which shares the same loci with *AtTUA2* and supposed to be a result of local duplication. *AtTUA2* (841425) has the same genomic context as it is ortholog in other species, having *TOR* (target of rapamycin, AT1G50030, 841427) gene on its 3`-side and the gene of ribosomal RNA small subunit methyltransferase E (AT1G50000, 841424) on 5`-side. Respectively, only *AlTUA2* (9330288) has the same genomic context with *TOR* and methyltransferase E located at both sides of the gene. In *C. sativa*, only *CsTUA2-A* (104742538), *CsTUA2-B* (104777925), *CsTUA2-C* (104758270) possess such genomic context, suggesting that these genes correspond to TUA2 isotype, while initially they were mistakenly annotated as the members of TUA6.

Genes *AtTUA4* and *AtTUA6* are found in the genome of *A. thaliana* at their unique loci, in which the majority of investigated orthologs of TUA Class I genes are not usually observed. *AtTUA4* gene is surrounded by Ypt/Rab-GAP domain of *gyp1p* superfamily protein gene (AT1G04830, 839404) on 5`-side and by Rpn2/Psmd1 subunit (AT1G04810, 839409) gene on 3`-side, while *AtTUA6* has *MAPR4* gene (AT4G14965, 827155) on 5`- and *KMS1* (AT4G14950, 827153) on 3`-side. No *TUA* genes were observed in such genomic context neither in *A. lyrata*, nor in *C. sativa* genomes. On contrary, *AlTUA4p* gene (possibly, the pseudogene of formerly functional *AlTUA4*) is surrounded by pre-mRNA-processing protein 40B (9321389) gene from 5`-side and by MADS-box protein *AGL62* gene (9319265) from 3`-side. Such genomic context for *TUA4* genes is also observed in number of other Brassicaceae species, beside *C. sativa*. *CsTUA4-A*, *CsTUA4-B* and *CsTUA4-C* are also flanked by pre-mRNA-processing protein 40B gene from 5`-side, similarly to *AlTUA4p*, what was the purpose to designate these genes as orthologs of *AlTUA4p*.

*AtTUA6*, *AlTUA6* and *CsTUA6-A*/*B*/*C* genes do not share similar genomic landscape. At the same time, the sequence identity among these genes seems to be not sufficient, what in combination with the previous fact suggests against possible orthology of the mentioned *TUA6* genes. For example, *AtTUA6* has the highest level of similarity with sequences of all three *CsTUA2* genes (99.56-99.78%), but *AtTUA6* and *CsTUA2*-*A*/*B*/*C* have different genomic context and, thus, are contained in non-homologous loci. It may be assumed that all *TUA6* genes may arose independently in *A. thaliana*, *A. lyrata* and in diploid progenitors of *C. sativa* via the series of independent duplications from *TUA2* or *TUA4*. Nevertheless, at this point of current investigation we propose to keep traditional name of *TUA6* for these genes (*AtTUA6*, *AlTUA6* and *CsTUA6-A*/*B*/*C*), since more species from Camelineae tribe are required to be analyzed, until the history of *TUA6* paralogy will be clarified.

Additionally, it is also important to say that *TUA6* does not represent a distinct isotype of TUA Class I, but rather being a group of independent paralogs. As it was mentioned above, orthologs of *AlTUA2* and *AlTUA4p* genes are present in the genomes of other Brassicaceae species, contrarily to *AlTUA6*, *AtTUA4* and *AtTUA6*. Genomes of two species were examined as the referent examples: *Descurainia sophioides* – as an example of close relative to Camelineae tribe from Brassicaceae Lineage I; and *Alyssum linifolium* – as a distant species, which belongs to the basal Brassicaceae Lineage. Both species possess relatively small genomes, suggesting that they have not faced numerous WGD- or allopolyploidy events, contrarily to *C. sativa*. TUA Class I genes are represented only with homologues of *AtTUA2*/*AlTUA2* (as well as *CsTUA2-A*, *CsTUA2-B*, *CsTUA2-C*) and *AlTUA4p* (and *CsTUA4-A*, *CsTUA4-B*, *CsTUA4-C*) genes in both genomes of *D. sophiodes* and *A. linifolium*.

Only four TUA genes from Class I are present in the genome of *A. linifolium*: two orthologs of *AtTUA2*/*AlTUA2*/*CsTUA2* – ohnologous (or possibly homeologous) *AliTUA2-A* and *AliTUA2-B* (Alyli.0070s0182 and Alyli.0075s0077, respectively). Both *AliTUA2-A* and *AliTUA2-B* were identified by similar genomic context of *AtTUA2*/*AlTUA2* (flanked by homologs of AT1G50000 (*TOR*) and AT1G50030 (methyltransferase E gene)), apart from sequence similarity with *AtTUA2*. Another two orthologs of *AlTUA4p*/*CsTUA4* are *AliTUA4-A* and *AliTUA4-B*, which are located near homologs of pre-mRNA-processing protein 40B (in *A. lyrata* - 9321389), similarly to *TUA4* genes in *A. lyrata* and *C. sativa*. Absence of the genomic landscape, typical for *AtTUA4* of *A. thaliana*, serves an additional evidence that current *AtTUA4* has paralogous relations with other *TUA* genes and likely represents the result of single gene duplication, accompanied by loss of actual *TUA4* at original locus.

In parallel, there are also only two TUA Class I genes in the genome of *D. sophioides*. The first one, *DsTUA2-1* (Desop.0030s0215.1), is an ortholog of all mentioned TUA2 genes of *A. thaliana*, *A. lyrata* and *C. sativa*. Respectively, *DsTUA2* is surrounded by homologs of AT1G50000 (Desop.0030s0214.1) and by AT1G50030 (Desop.0030s0217.1). However, it is interesting to mention that *DsTUA2-1* has potentially paralogous pseudogene, *DsTUA2p-2* (Desop.0030s0216.1), on its 3`-side, which is inverted in relation to the functional gene. *AtTUA2p* demonstrates similar allocation in the genome of *A. thaliana*, as well as *DsTUA2p-2*. *DsTUA2p-2* possesses high similarity to the *AtTUA2p* (AT1G50020) in *A. thaliana* genome – 94.2 %, which may suggest about orthologous relation between *DsTUA2p-2* and *AtTUA2p*. The most interesting fact is that both *AliTUA2-A* and *AliTUA2-B* have same paralogous pseudogenes at their loci - *AliTUA2p-A* (Alyli.0070s0183) and *AliTUA2p-B* (Alyli.0075s0078), which are also possess 99% of similarity to *DsTUA2p-2*. These facts might suggest that *AtTUA2p* is an ancient pseudogene. The reasons for conservancy of this pseudogene in such distant species are currently unknown. However, orthologs of these pseudogenes have been completely lost in the genomes of *A. lyrata* and *C. sativa*.

Only one gene, orthologous to *AlTUA4* and *CsTUA4-A*/*B*/*C*, was detected within the genome of *D. sophioides* – *DsTUA4* (Desop.0049s0018.1). It shares typical loci for *TUA4* and being surrounded with homolog of pre-mRNA-processing protein 40B gene from 5`-side (Desop.0049s0019.1) and by *AGL62* from 3`-side (Desop.0049s0017.1), respectively. Moreover, no orthologs of so-called *TUA6* were identified within the genomes of *D. sophiodes* and *A. linifolium*, additionally suggesting that all *TUA6* of Camelineae species may have paralogous nature.

**Table S1.** Corrected *TUA* gene names, used in the present study

| Gene name used in present study (Proposed name) | Gene ID | Organism | Annotation name of isotype (by analogy with *A. thaliana*) |
| --- | --- | --- | --- |
| *AtTUA1* | 842782 | *A. thaliana* | TUA1 |
| *AtTUA2* | 841425 |  | TUA2 |
| *AtTUA2p* | 841426 |  | TUA6 |
| *AtTUA3* | 832097 |  | TUA3 |
| *AtTUA4* | 839405 |  | TUA4 |
| *AtTUA5* | 832098 |  | TUA5 |
| *AtTUA6* | 827154 |  | TUA6 |
| *AlTUA1* | 9324341 | *A. lyrata* | TUA1 |
| *AlTUA2* | 9330288 |  | TUA2 |
| *AlTUA3* | 9310030 |  | TUA3 |
| *AlTUA4p* | 9321390 |  | TUA2 |
| *AlTUA5* | 9310031 |  | TUA3 |
| *AlTUA6* | 9299671 |  | TUA2 |
| *AliTUA2-A* | Alyli.0070s0182 | *Alyssum linifolium* | TUA2 |
| *AliTUA2-B* | Alyli.0075s0077 |  | TUA2 |
| *AliTUA2p-A* | Alyli.0070s0183 |  | TUA6 |
| *AliTUA2p-B* | Alyli.0075s0078 |  | TUA6 |
| *AliTUA4-A* | Alyli.0029s0065 |  | TUA2 |
| *AliTUA4-B* | Alyli.0204s0039 |  | TUA2 |
| *DsTUA2-1* | Desop.0030s0215.1 | *Descurainia sophioides* | TUA2 |
| *DsTUA2p-2* | Desop.0030s0216.1 |  | TUA6 |
| *DsTUA4* | Desop.0049s0018.1 |  | TUA2 |

**Table S2.** Names and ID of TUA protein sequences, used for ML tree construction

| Species | Protein name | Sequence ID* |
| --- | --- | --- |
| *Betula pendula* | BpTUA | CAB66336 |
| *Gossypium hirsutum* | GhTUA1 | AAQ92661 |
|  | GhTUA2 | AAQ92662 |
|  | GhTUA4 | AAQ92663 |
| *Prunus dulcis* | PdTUA1 | CAA47635 |
| *Hordeum vulgare* | HvTUA1 | CAA67942 |
|  | HvTUA2 | CAA69724 |
|  | HvTUA3 | CAA10663 |
| *Pseudotsuga menziesii* | PmTUA1 | AAV92379 |
| *Oryza sativa* | OsTUA1 | Os03g0726100 |
|  | OsTUA2 | Os11g0247300 |
|  | OsTUA3 | Q0PVB0 |
|  | OsTUA4 | Q10PW2 |
| *Eleusine indica* | EiTUA1 | CAA06618 |
|  | EiTUA2 | O22348 |
|  | EiTUA3 | O22349 |
| *Zea mays* | ZmTUA1 | CAA33734 |
|  | ZmTUA2 | CAA33733 |
|  | ZmTUA3 | CAA44861 |
|  | ZmTUA5 | CAA44862 |
|  | ZmTUA6 | CAA44863 |
| *Solanum tuberosum* | StTUA1 | ABB02631 |
|  | StTUA2 | ABB16994 |
| *Setaria viridis* | SvTUA1 | CAE52514 |
|  | SvTUA2 | CAE52515 |
| *Eucalyptus grandis* | EgTUA1 | A7KQH5 |
|  | EgTUA3-1 | A0A059D9I7 |
|  | EgTUA3-2 | A0A059DHJ6 |
|  | EgTUA-1 | A0A059BLE3 |
|  | EgTUA-2 | A0A059BC05 |
| *Populus tremuloides* | PtTUA1 | AY229881 |
|  | PtTUA2 | AY229882 |
|  | PtTUA3 | EF583813 |
|  | PtTUA4 | EF584828 |
|  | PtTUA5 | EF583814 |
|  | PtTUA6 | EF583816 |
|  | PtTUA7 | EF584829 |
| *Linum usitatissimum* | LuTUA1 | Lus10035422 |
|  | LuTUA2 | Lus10031032 |
|  | LuTUA3 | Lus10013765 |
|  | LuTUA4 | Lus10039169 |
|  | LuTUA5 | Lus10020281 |
|  | LuTUA6 | Lus10005705 |
| *Arabidopsis thaliana* | AtTUA1 | P11139 |
|  | AtTUA2 | B9DGT7 |
|  | AtTUA3 | Q56WH1 |
|  | AtTUA4 | Q0WV25 |
|  | AtTUA5 | B9DHQ0 |
|  | AtTUA6 | P29511 |
| *Arabidopsis lyrata* | AlTUA6 | XP_020884005 |
| *Camelina sativa* | CsTUA2-A | XP_010461854 |
|  | CsTUA2-B | XP_010500555 |
|  | CsTUA2-C | XP_010479400 |
|  | CsTUA4-A | XP_010487911 |
|  | CsTUA4-B | XP_010507910 |
|  | CsTUA4-C | XP_010466126 |
|  | CsTUA6-A | XP_010418156 |
|  | CsTUA6-B | XP_010473400 |
|  | CsTUA6-C | XP_010430201 |
|  | CsTUA1-A | XP_010414865 |
|  | CsTUA1-Un | XP_010496498 |
|  | CsTUA3-A | XP_010420793 |
|  | CsTUA3-B | XP_010454260 |
|  | CsTUA3-C | XP_010493056 |
|  | CsTUA5-B | XP_010454262 |
|  | CsTUA5-C | XP_010493058 |
| *Chlamydomonas reinhardtii* | ChlR_TUA1 | P09204 |
|  | ChlR_TUA2 | P09205 |

* Phytozome, NCBI and UniProt IDs

**Identification, characterization and isotype classification of the identified β-tubulins**


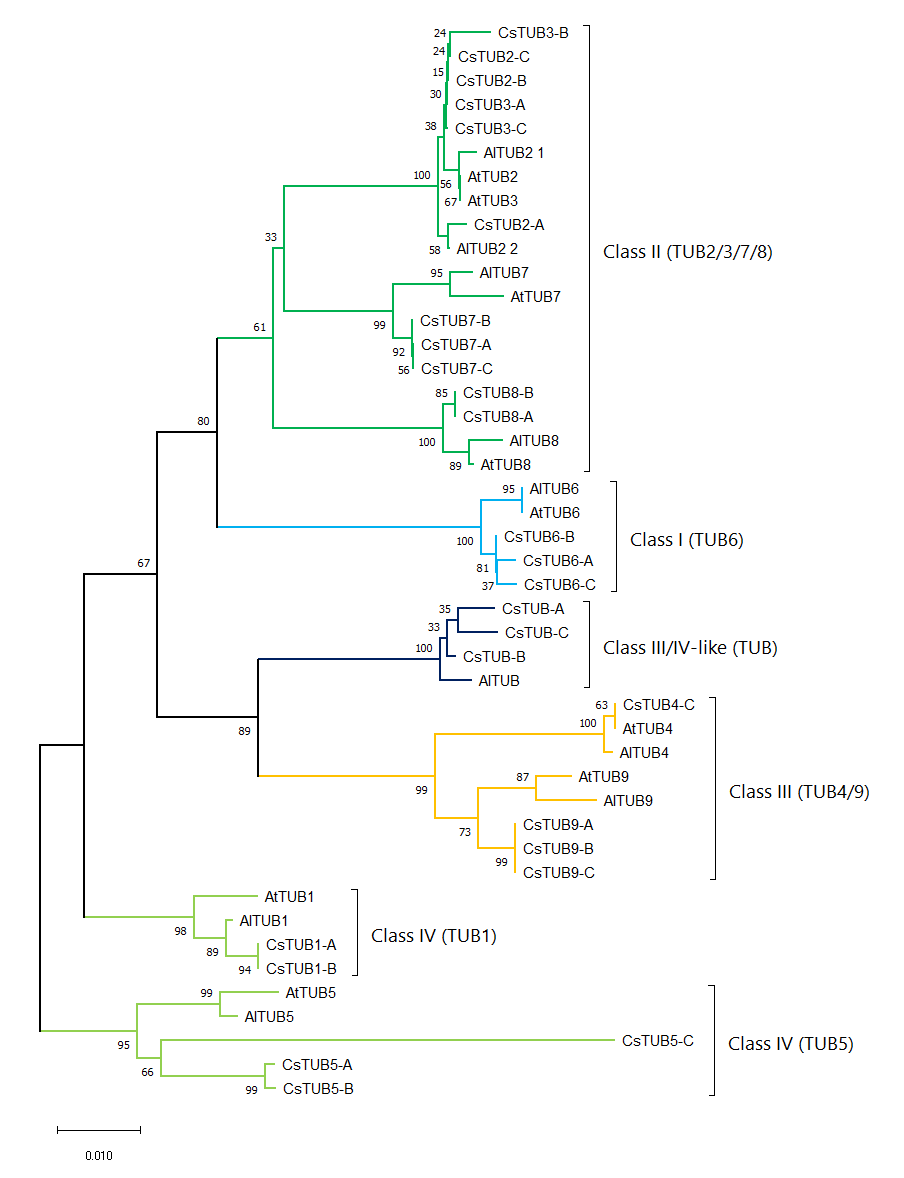


**Figure S4.** Phylogenetic tree (NJ) of *A. thaliana*, *A. lyrata* and *C. sativa* TUB protein sequences with 1000 replicates bootstrap support. Initial identification of TUB isotypes was conducted, basing on the results of this analysis.


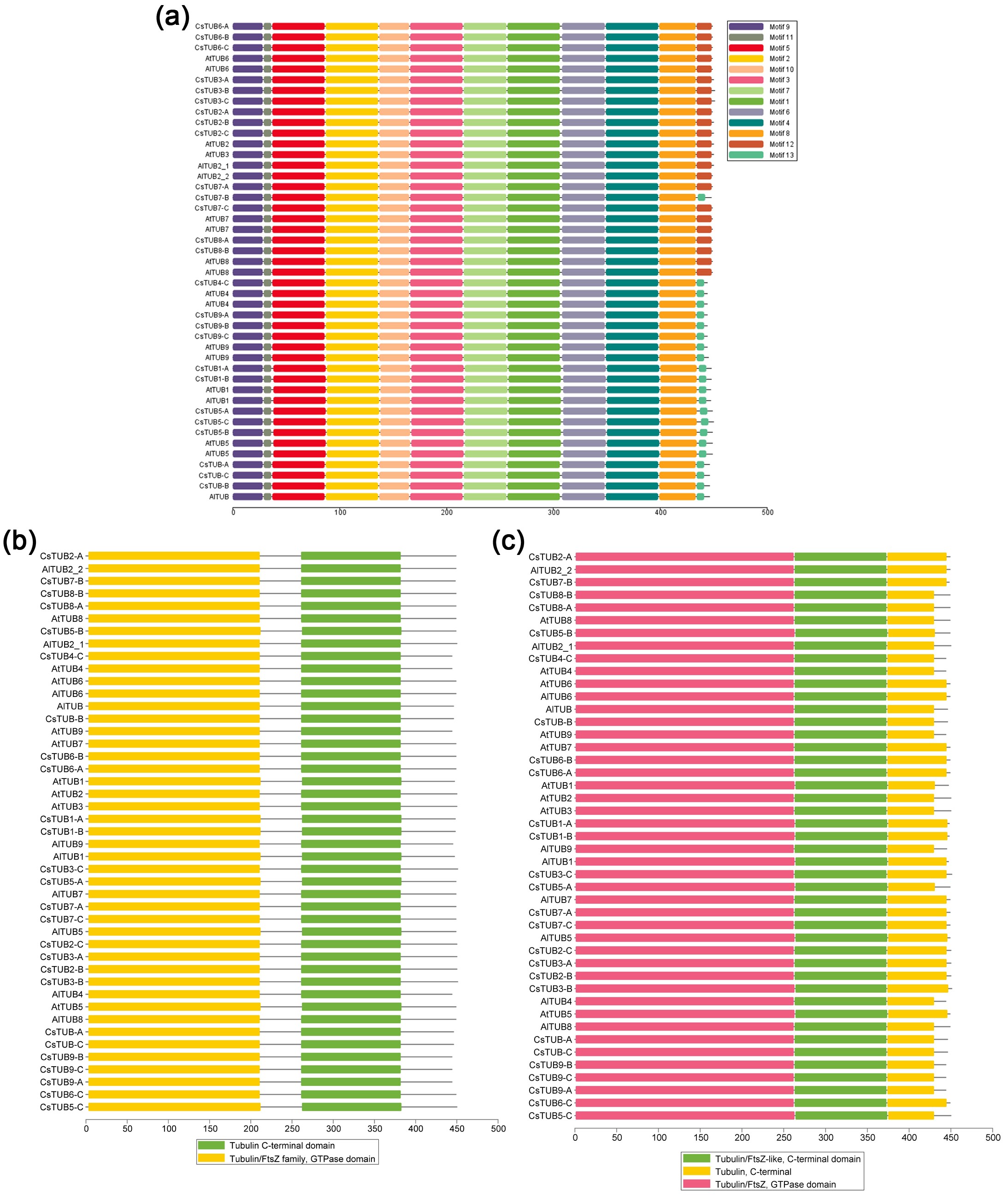


**Figure S5.** Distribution of conserved sequence motifs and functional domains within the identified β-tubulin peptides. **(a)** Conserved peptide sequence motifs in the identified β-tubulins; **(b)** functional domains of the β-tubulins identified against Pfam database; **(c)** structural domains identified against CATH-Gene3D database.

**Supplementary Note 2.** Loci differences within the β-tubulin genes of *C. sativa* and related Brassicaceae species

**Genomic landscape of TUB2 and TUB3 loci in *C. sativa* and related Brassicaceae species**

It was not possible to distinguish correctly TUB2 and TUB3 isotypes, based on sequences comparison, since the genes of these isotypes encode almost similar proteins (99.11-100%) with only few different residues, which are, obviously, not enough to distinguish these isotypes. Therefore, we investigated genomic landscape differences at the loci of TUB2/3, in order to diversify these genes and identify their exact orthologous relations, as it was done in the case of α-tubulins.

The *AtTUB3* has gene of leucine-rich repeat kinase family protein, or LRR receptor-like serine/threonine-protein kinase FEI 1 (AT5G62710, *A. thalina* GeneID – 836392, *A. lyrata* – 9300889) next to its 3`-end. Moreover, in the *A. thaliana* genome, *AtTUB2* and *AtTUB3* genes are located one after another and encode identical proteins. It is likely that TUB2 and TUB3 isotypes arose as a result of local gene duplication event during the speciation of *A. thaliana*. On contrary, TUB2/3 isotype is represented by two genes in *A. lyrata* genome. Only one copy of *AlTUB2-1* (corrected gene name - *AlTUB3*) presents at locus (*FEI 1* and *AtTUB3* allocation) homologous to *A. thaliana* ones. The other gene, *AlTUB2-2* (*AlTUB2*, 9311881), is present in a different genomic region, near two At3g47150 homologs (9313587 and 9311880) that are located at the 3`-end of *AlTUB2-2*. At the same time, no orthologous gene of *AlTUB3* is present at At3g47150 locus in *A. thaliana* genome. Taking in account the described peculiarities, we propose to designate β-tubulin genes gene, related to *FEI 1* loci, as members of a distinct isotype (orthologous lineage) of *TUB3.* Therefore, the orthologs of *AlTUB2-1* (*AlTUB3*) and *AtTUB2/3* (*AtTUB3-1* and *AtTUB3-2*) should be viewed as the members of *TUB2* isotypes (the list of this corrected gene nomenclature is provided in Table S3).

Presence of distinct TUB2 and TUB3 isotypes is not limited only to Camelineae species. Both *DsTUB2* (Desop.0207s0573, flanked by orthologs of AT3G47130, AT3G47140, AT3G47150) and *DsTUB3* (Desop.0011s0165 located the ortholog of *LRR*-kinase AT5G62710) were identified at their expected loci in the genome of *D. sophioides*. Similarly, two copies of *AliTUB2-A* (Alyli.0002s0222) and *AliTUB2-B* (Alyli.0056s0114) and one functional *AliTUB3-A* gene (Alyli.0117s0072, second ohnologous copy seems to be lost or pseudogenised) were identified within the genome of *A. linifolium* at their loci expected genomic context (Table S3). Therefore, it can be concluded that the case of *A. thaliana* with the loss of ‘true *AtTUB2*’ and the duplication of *AtTUB3* seems to be inherent for this particular species only.

Similarly, the genes TUB2 and TUB3 were distinguished in *C. sativa*, based on their genomic context differences. All *CsTUB2* and *CsTUB3* homeologs are contained in separate loci and located on different chromosomes within all three *C. sativa* subgenomes. In particular, genes *CsTUB3-A* (104726871), *CsTUB3-B* (104762402) and *CsTUB3-C* (104740363) were always co-located with *FEI 1* on their 3’ side. Contrarily, genes *CsTUB2-A* (104780498), *CsTUB2-B* (104790913) and *CsTUB2-C* (104711158) are present on chromosome, different from the TUB3 genes location and were observed in typical TUB2 genomic context, alike in *A. lyrata*. *CsTUB2* homelogs were also flanked (on 3`-end) by pseudogenes, homologous to At3g47150 F-box protein (in A-subgenome – 104783859, B – 104698929 and C – 104715351).

**Genomic landscape of TUB10 locus in *C. sativa* and related Brassicaceae species**

Both *AlTUB* (*AlTUB10*) and *CsTUB-A*/*B*/*C* genes are located upstream to mRNA-RNA polymerase I-specific transcription initiation factor (*RRN3*) gene (9315581, in the genome of *A. lyrata*). In parallel, genes of this isotype in other referent species, such as *D. sophiodes* (*DsTUB*, Desop.0228s0387) and *A. linifolium* (*AliTUB-A*, Alyli.0025s0123; *AliTUB-B*, Alyli.0603s0023) were found at the same loci with *RRN3* homologs (Table S3). No other β-tubulin genes were observed at such loci.

**Table S3.** Novel, corrected or altered *TUB* gene names, used in the present study

| Gene name used in present study (Proposed name) | Gene ID | Organism | Annotation name of isotype (by analogy with *A. thaliana*) |
| --- | --- | --- | --- |
| *AtTUB3-1*  (AtTUB2)* | 836390  AT5G62690 | *A. thaliana* | TUB2 |
| *AtTUB3-2*  (AtTUB3)* | 836391  AT5G62700 |  | TUB2 |
| *AlTUB*  (*AlTUB10*) | 9317423 | *A. lyrata* | TUB |
| *AlTUB3*  (*AlTUB2-1*) | 9302580 |  | TUB2 |
| *AlTUB2*  (*AlTUB2-2*) | 9311881 |  | TUB2 |
| *AliTUB-A*  (*AliTUB10-A*) | Alyli.0025s0123 | *A. linifolium* | TUB |
| *AliTUB-B*  (*AliTUB10-B*) | Alyli.0603s0023 |  | TUB |
| *AliTUB2-A* | Alyli.0002s0222 |  | TUB2 |
| *AliTUB2-B* | Alyli.0056s0114 |  | TUB2 |
| *AliTUB3-A* | Alyli.0117s0072 |  | TUB2 |
| *DsTUB*  (*DsTUB-10*) | Desop.0228s0387 | *D. sophioides* | TUB |
| *DsTUB2* | Desop.0207s0573 |  | TUB2 |
| *DsTUB3* | Desop.0011s0165 |  | TUB2 |

* - current name of gene


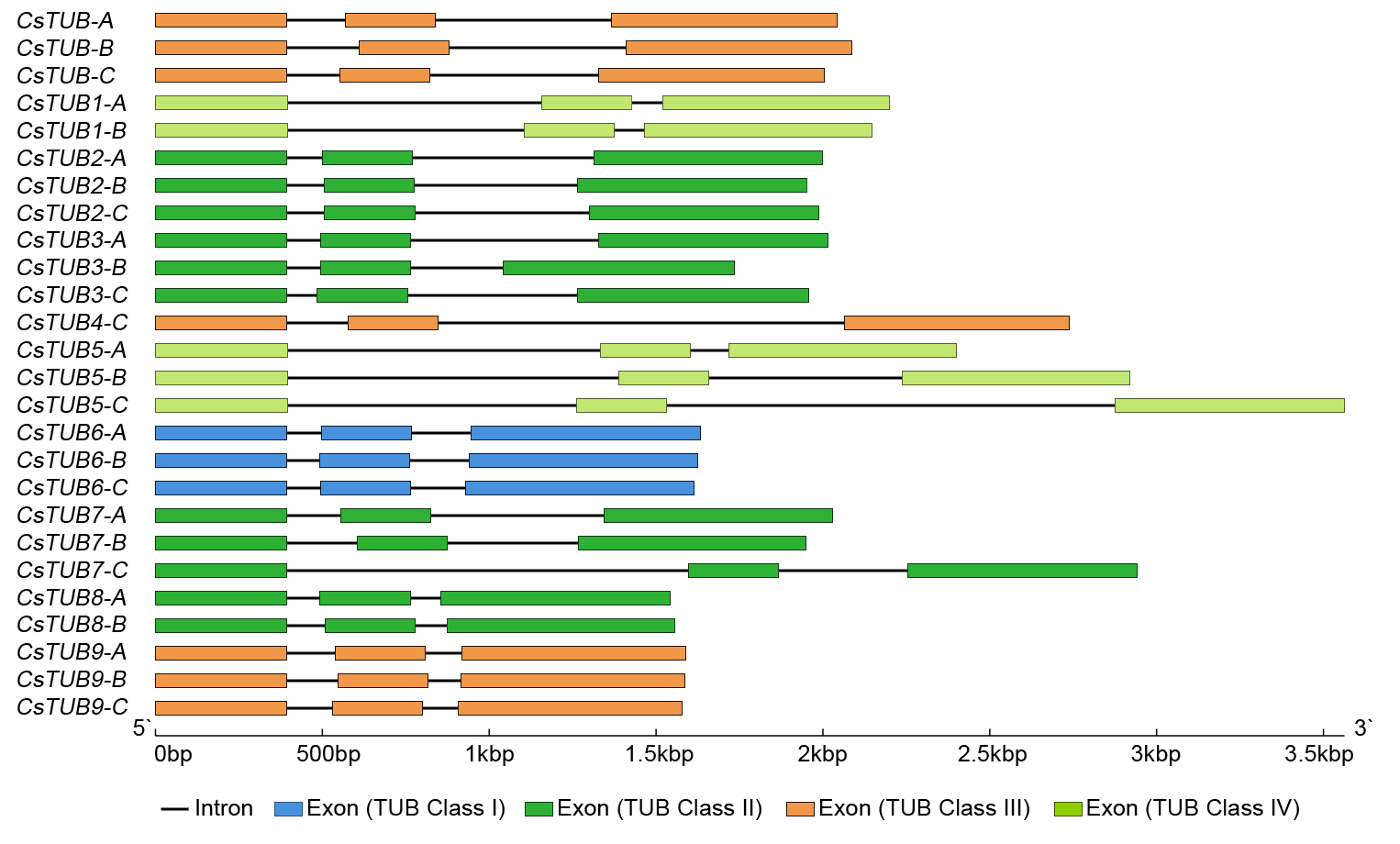


**Figure S6.** Exon-intron structure of identified β-tubulin genes in *C. sativa* genome.

**Table S4.** Names and ID of TUB protein sequences, used for ML tree construction

| Species | Protein name | Sequence ID* |
| --- | --- | --- |
| *Oryza sativa* | OsTUB1 | Os01g0282800 |
|  | OsTUB2 | Os03g0105600 |
|  | OsTUB3 | Os06g0671900 |
|  | OsTUB4 | Os01g0805900 |
|  | OsTUB5 | Os02g0167300 |
|  | OsTUB6 | Os05g0413200 |
|  | OsTUB7 | Os03g0780600 |
|  | OsTUB8 | Os03g0661300 |
| *Zea mays* | ZmTUB1 | CAA37060 |
|  | ZmTUB2 | CAA37061 |
|  | ZmTUB3 | CAA52718 |
|  | ZmTUB4 | CAA52719 |
|  | ZmTUB5 | CAA52720 |
|  | ZmTUB6 | Q41783 |
|  | ZmTUB7 | Q41784 |
|  | ZmTUB8 | Q41785 |
| *Zinnia elegans* | ZeTUB1 | BAA82637 |
|  | ZeTUB2 | BAA82638 |
| *Eucalyptus grandis* | EgTUB1 | EF534219 |
|  | EgTUB2 | EF534220 |
|  | EgTUB3 | EF534221 |
|  | EgTUB4 | EF534222 |
|  | EgTUB5 | EF534223 |
| *Lycopersicon esculentum* | LeTUB1 | BT013153 |
|  | LeTUB2 | BT014148 |
|  | LeTUB3 | BT012803 |
|  | LeTUB4 | DQ205342 |
|  | LeTUB5 | BT013893 |
|  | LeTUB6 | BT013141 |
| *Solanum tuberosum* | StTUB1 | CAA83847 |
|  | StTUB2 | CAA83853 |
|  | StTUB3 | ABA46773 |
|  | StTUB4 | ABA81852 |
| *Linum usitatissimum* | LusTUB1a | KM196480 |
|  | LusTUB1b | KM196481 |
|  | LusTUB2a | KM196482 |
|  | LusTUB2b | KM196483 |
|  | LusTUB3a | KM196484 |
|  | LusTUB3b | KM196485 |
|  | LusTUB4a | KM196486 |
|  | LusTUB4b | KM196487 |
|  | LusTUB5 | KM196488 |
|  | LusTUB6a | KM196489 |
|  | LusTUB6b | KM196490 |
|  | LusTUB7a | KM196491 |
|  | LusTUB7b | KM196492 |
|  | LusTUB7c | KM196493 |
| *Prunus persica* | PpTUB1 (PpTUB-5) | XP_007222097 |
|  | PpTUB2 (PpTUB1) | XP_007218003 |
|  | PpTUB3 (PpTUB-4) | XP_007211629 |
|  | PpTUB4 (PpTUB-3) | XP_007211186 |
|  | PpTUB5 (PpTUB-2) | XP_007209134 |
|  | PpTUB6 (PpTUB-6) | XP_020423113 |
|  | PpTUB7 (PpTUB-1) | XP_007205196 |
|  | PpTUB8 (PpTUB5) | XP_020423871 |
| *Medicago truncatula* | MtrTUB1** | XP_013457308.1 |
|  | MtrTUB2** | XP_003631111.1 |
|  | MtrTUB3** | XP_003630513.1 |
|  | MtrTUB4** | XP_003604818.1 |
|  | MtrTUB5** | XP_003604816.1 |
|  | MtrTUB6** | XP_003624926.1 |
|  | MtrTUB7** | XP_003603765.1 |
|  | MtrTUB8** | XP_003594596.1 |
|  | MtrTUB9** | XP_003592284.1 |
| *Populus trichocarpa* | PtrTub1 | XP_002298330.1 |
|  | PtrTub2 | XP_002313404.2 |
|  | PtrTub3 | XP_002299541.1 |
|  | PtrTub4 | XP_002304488.1 |
|  | PtrTub5 | XP_002309109.1 |
|  | PtrTub6 | XP_002323545.3 |
|  | PtrTub7 | XP_002299042.1 |
|  | PtrTub8 | XP_002330612.1 |
|  | PtrTub9 | XP_002298036.2 |
|  | PtrTub10 | XP_002304492.1 |
|  | PtrTub11 | XP_002318519.1 |
|  | PtrTub12 | XP_002321512.2 |
|  | PtrTub13 | XP_002298454.1 |
|  | PtrTub14 | XP_002314014.3 |
|  | PtrTub16 | XP_002314013.1 |
|  | PtrTub17 | XP_002307960.1 |
|  | PtrTub18 | XP_002322609.1 |
|  | PtrTub19 | XP_002300703.1 |
|  | PtrTub20 | XP_002307727.1 |
| *Lactuca sativa* | LsTUB1** | A0A2J6K0J6 |
|  | LsTUB2** | A0A2J6KDB4 |
|  | LsTUB3** | A0A2J6KIE2 |
|  | LsTUB4** | A0A2J6KUF4 |
|  | LsTUB5** | A0A2J6KVA4 |
|  | LsTUB6** | A0A2J6L4W4 |
|  | LsTUB7** | A0A2J6L9G6 |
|  | LsTUB8** | A0A2J6LP40 |
|  | LsTUB9** | A0A2J6M1U4 |
|  | LsTUB10** | A0A2J6M466 |
|  | LsTUB11** | A0A2J6M4F4 |
| *Citrus clementina* | CcTUB1** | V4SZ36 |
|  | CcTUB2** | V4T2M6 |
|  | CcTUB3** | V4T5E8 |
|  | CcTUB4** | V4THG6 |
|  | CcTUB5** | V4U7G6 |
|  | CcTUB6** | V4U815 |
|  | CcTUB7** | V4UFX5 |
| *Arabidopsis thaliana* | AtTUB1 | P12411 |
|  | AtTUB2 | Q56YW9 |
|  | AtTUB3 | Q9ASR0 |
|  | AtTUB4 | P24636 |
|  | AtTUB5 | P29513 |
|  | AtTUB6 | P29514 |
|  | AtTUB7 | P29515 |
|  | AtTUB8 | P29516 |
|  | AtTUB9 | P29517 |
| *Arabidopsis lyrata* | AlTUB | D7LH43 |
| *Camelina sativa* | CsTUB6-A | XP_010419814 |
|  | CsTUB6-B | XP_010453291 |
|  | CsTUB6-C | XP_010491982 |
|  | CsTUB2-A | XP_010503303 |
|  | CsTUB2-B | XP_010515012 |
|  | CsTUB2-C | NP_001289924 |
|  | CsTUB3-A | XP_010444122 |
|  | CsTUB3-B | XP_010483981 |
|  | CsTUB3-C | XP_010459234 |
|  | CsTUB7-A | XP_010414372 |
|  | CsTUB7-B | XP_010469941 |
|  | CsTUB7-C | XP_010510475 |
|  | CsTUB8-A | XP_010421190 |
|  | CsTUB8-B | XP_010454670 |
|  | CsTUB4-C | XP_010494444 |
|  | CsTUB9-A | XP_010439552 |
|  | CsTUB9-B | XP_010434259 |
|  | CsTUB9-C | XP_010449142 |
|  | CsTUB1-A | XP_010416482 |
|  | CsTUB1-B | XP_010471708 |
|  | CsTUB5-A | XP_010459680 |
|  | CsTUB5-B | XP_010498429 |
|  | CsTUB5-C | XP_010477226 |
|  | CsTUB-A | XP_010504960 |
|  | CsTUB-B | XP_010516638 |
|  | CsTUB-C | XP_010509706 |
| *Chlamydomonas reinhardtii* | ChlRTUB1/2 | P04690.1 |

* Phytozome, NCBI and UniProt IDs

** Technical name assigned in the present study

**Supplementary Note 3.** Characterization of β-tubulin pseudogenes

The presence of numerous copies of highly conserved β-tubulin genes in *C. sativa* genome might lead to elimination of one or two homeologous copies of a certain isotype. Unlike the situation in α-tubulin subfamily, the genes of at least three β-tubulin isotypes were found to be pseudogenic, involving one or two homeologous copies (Table S5). In particular, third copy of TUB1 gene is pseudogenised in the C subgenome (*CsTUB1p-C*), as well as *CsTUB8p-C* that also faced the same fate. Both of these pseudogenes are located in regions, homologous to those of their functional copies from A and B subgenomes.

The genome of *C. sativa* contains three pseudogenes of TUB4 isotype and only one functional gene – *CsTUB4-C*. At the same time, *CsTUB4p-C* is located on different chromosome from *CsTUB4-C*, excluding the possibility of the pseudogene origin via any kind of tandem duplication. *CsTUB4p-C* is located on non-homologous chromosome (Chr12), compared to other TUB4 genes (Chr18 in C subgenome and Chr11 in A). Surprisingly, *CsTUB4p-C* also does not share same genomic context as *CsTUB4p-A*, *CsTUB4p-B*, *CsTUB4-C*, which all have *Pollenless 3-like 1* (the homolog of AT5G44330) on their 3`-sides. Thus, the origin of *CsTUB4p-C* is unclear, and possibly caused by non-homologous recombination or another process.

The origins of *CsTUB3p-C*, *CsTUB9p-A* and *CsTUB9p-B* seem to be clearly paralogous, since these pseudogenes are very close to their functional homologs. In particular, *CsTUB9p-A* and *CsTUB9p-B* are located at the same loci as *CsTUB9-A* and *CsTUB9-B* respectively. *CsTUB3p-C* is located 0.5 Mbp downstream from the functional *CsTUB3-C* and does not share the same genomic context, suggesting this pseudogene may result from a single gene duplication event.

**Table S5. The identified potential β-tubulin pseudogenes**

| Proposed gene name | NCBI gene ID | Gene length  (bp) | Exons | Peptide length (aa) | Aberration type | Chr and sub-genome |
| --- | --- | --- | --- | --- | --- | --- |
| *CsTUB3p-C* | 104756937 | 1759 | 4 | 308 | Duplication of *CsTUB3-C*; partial loss of exon3; disruption of exon1 | 2, G3 |
| *CsTUB8p-C* | 104772499 | 1444 | 3 | 353 | Partial loss of exon1 | 20, G3 |
| *CsTUB4p-A* | 104725007 | 1929 | (3) | - | Frameshift in exon1 (del255T) | 11, G1 |
| *CsTUB4p-B* | 104760508 | 1645 | 3 | 379 | Partial loss of exon1 | 18, G2 |
| *CsTUB4p-C* | 104732380 | 1777-1817 | 3-4 | 144 | Partial loss of exon1 and exon2; complete loss of exon3, disruption of exon2; expression of miscRNA | 12, G3 |
| *CsTUB9p-A* | 104722924 | 852 | 4 | 213 | Duplication of *CsTUB9-A*; loss of exon3; partial disruption of exon1 and exon2 | 11, G1 |
| *CsTUB9p-B* | 104718248 | 544 | 1 | 132 | Duplication of *CsTUB9-B*; complete loss of exon3 and exon 2 (except 4-nucleotide -GTTAG- fragment); | 10, G2 |
| *CsTUB1p-C* | 104758294 | 3364 | 4 | 407 | Partial loss of exon1 | 17, G3 |

**Identification, characterization and isotype classification of the identified γ-tubulins**


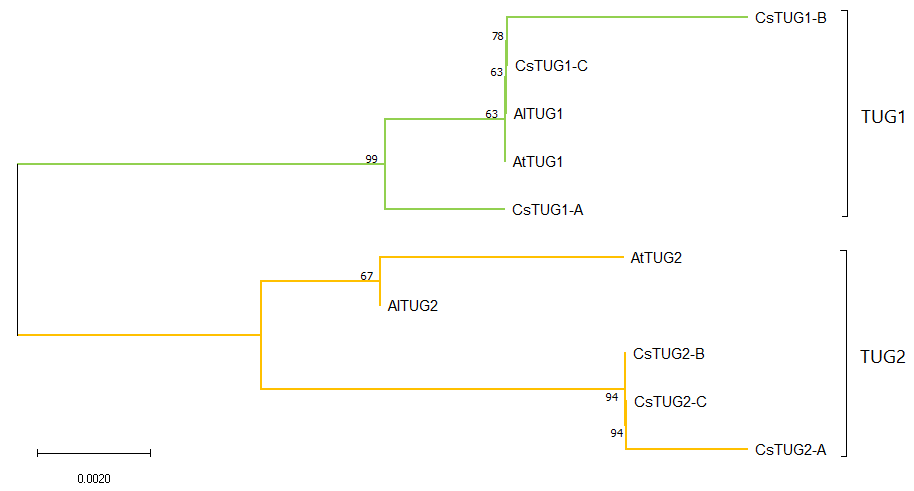


**Figure S7.** Phylogenetic tree (NJ) of *A. thaliana*, *A. lyrata* and *C. sativa* TUG protein sequences with 1000 replicates bootstrap support. Initial identification of TUG isotypes was conducted, basing on the results of this analysis.


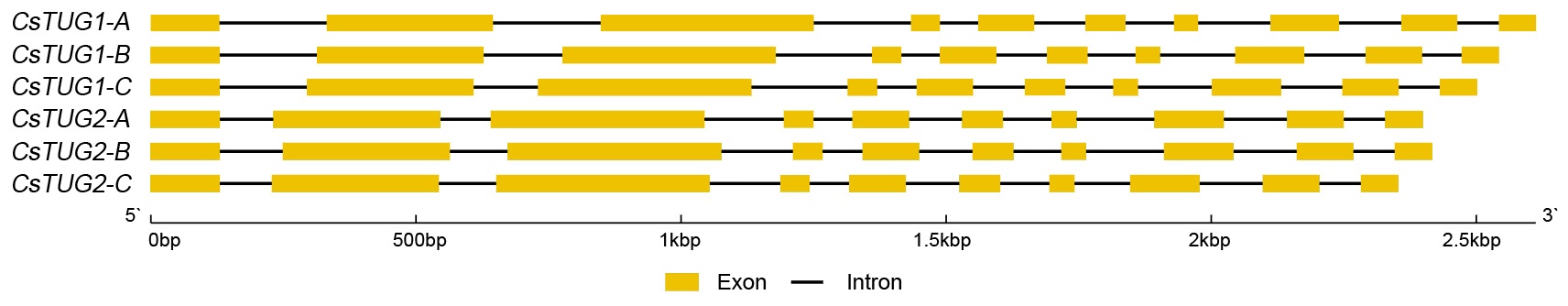


**Figure S8.** Intron-exon structure of identified γ-tubulin genes within the genome of *C. sativa*.


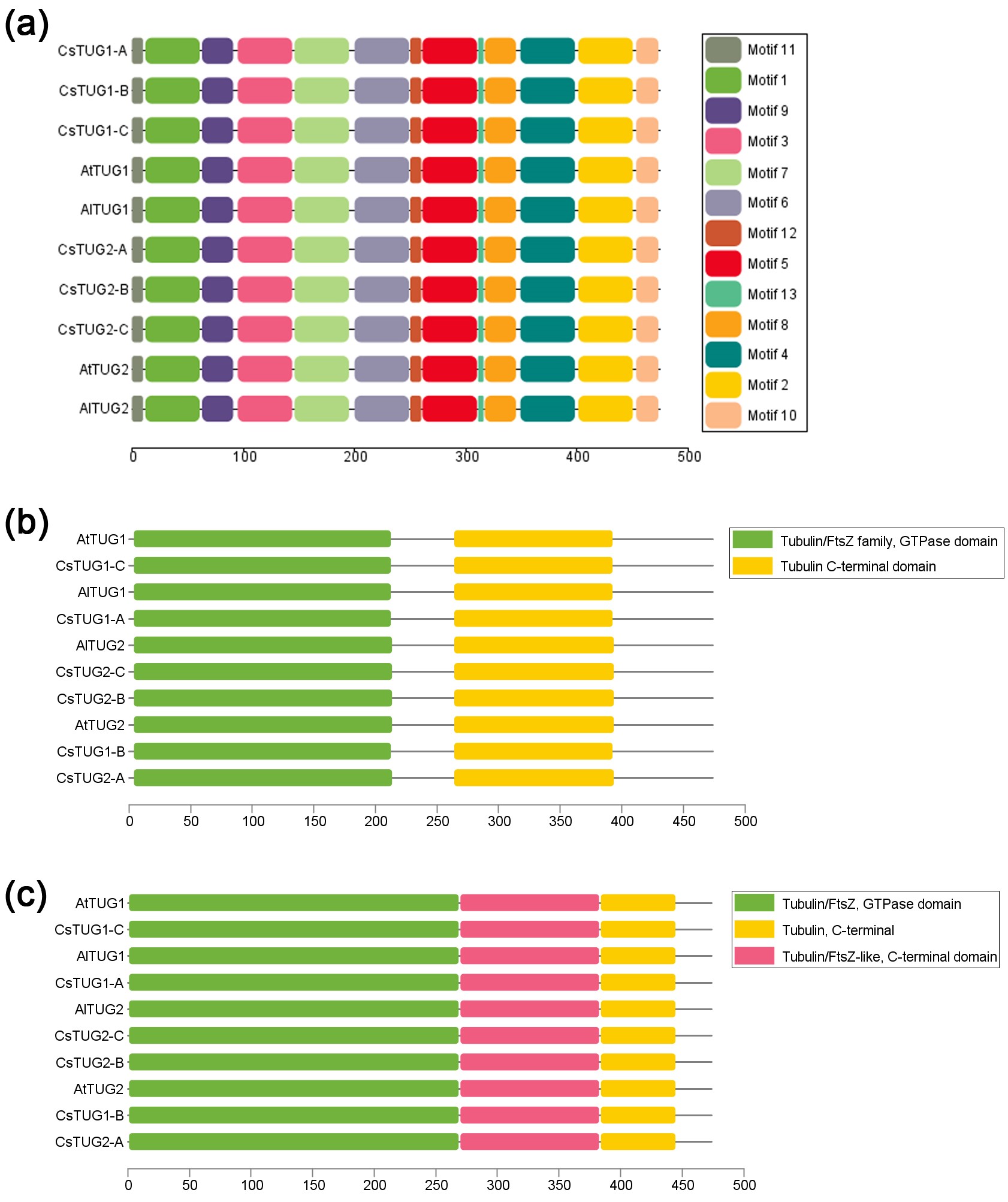


**Figure S9.** Distribution of conserved sequence motifs and functional domains within the identified γ-tubulin peptides. **(a)** Conserved peptide sequence motifs in the identified γ-tubulins; **(b)** functional domains of the γ-tubulins identified against Pfam database; **(c)** structural domains identified against CATH-Gene3D database.

**Table S6.** Names and ID of TUG protein sequences, used for ML tree construction

| Species | Protein name | Sequence ID* |
| --- | --- | --- |
| Solanum tuberosum | StTUG | M1B807 |
| Eucalyptus grandis | EgTUG | A0A059BZZ0 |
| Populus trichocarpa | PtTUG1 | B9I8U5 |
|  | PtTUG2 | A0A2K2BK21 |
| Oryza sativa subsp. japonica | OsTUG2 | O49068 |
| *Zea mays* | ZmTUG1 | Q41807 |
|  | ZmTUG2 | Q41808 |
|  | ZmTUG3 | Q41874 |
| Gossypium hirsutum | GhTUG1 | Gohir.A12G110800.1.p |
|  | GhTUG2 | Gohir.D12G114100.1.p |
|  | GhTUG3 | Gohir.A11G094800.1.p |
|  | GhTUG4 | Gohir.D11G099800.1.p |
| Triticum aestivum | TaTUG_B | Traes_1BS_2A33D289F.1 |
|  | TaTUG_D | Traes_1DS_1D69583A1.2 |
| Helianthus annuus | HaTUG1 | HanXRQChr15g0472351 |
|  | HaTUG2 | HanXRQChr04g0120991 |
| Glycine max | GmTUG1 | I1JRY0 |
|  | GmTUG2 | I1NCI3 |
| *Miscanthus sinensis* | MsTUG1 | Misin16G060500 |
|  | MsTUG2 | Misin17G065600 |
| *Medicago truncatula* | MtTUG | Medtr7g117050.1 |
| *Beta vulgaris* | BvTUG | EL10Ac1g00313.1 |
| *Solanum lycopersicum* | SlTUG | Solyc03g111380.3.1 |
| *Lactuca sativa* | LsTUG1 | Lsat_1_v5_gn_5_84941.1 |
|  | LsTUG2 | Lsat_1_v5_gn_7_1921.1 |
| *Salix purpurea* | SpTUG1 | Sapur.014G073600.1.p |
|  | SpTUG2 | Sapur.002G146800.1.p |
| *Theobroma cacao* | TcTUG | Thecc.01G347400.1.p |
| *Panicum virgatum* | PvTUG1 | Pavir.3NG220026.1.p |
|  | PvTUG2 | Pavir.3KG173700.1.p |
| *Setaria italica* | SiTUG | Seita.7G268800.1.p |
| *Thlaspi arvense* | ThaTUG | Thlar.0021s0186.1.p |
| *Eleusine coracana* | EcTUG | ELECO.r07.5AG0404110.1 |
| *Sorghum bicolor* | SbTUG | Sobic.009G052100.1.p |
| *Amaranthus hypochondriacus* | AhTUG | AH009599-RA |
| *Coffea arabica* | CaTUG1 | evm.model.Scaffold_633.864 |
|  | CaTUG2 | evm.model.Scaffold_2658.99 |
| *Manihot esculenta* | MeTUG1 | Manes.05G025900.1.p |
| *Malus domestica* | MdTUG1 | MD01G1076100 |
|  | MdTUG2 | MD07G1145100 |
| *Asparagus officinalis* | AoTUG1 | evm.model.AsparagusV1_02.2091 |
|  | AoTUG2 | evm.model.AsparagusV1_08.3137 |
| *Citrus sinensis* | CsinTUG | orange1.1g046749m |
| *Citrus clementina* | CcTUG | Ciclev10019995m |
| *Daucus carota* | DcTUG | DCAR_006965 |
| *Musa acuminata* | MaTUG | GSMUA_Achr1P14750_001 |
| *Arabidopsis halleri* | ArhTUG | Araha.13672s0002.1.p |
| *Cakile maritima* | CmTUG1 | Camar.0493s0030.1.p |
|  | CmTUG2 | Camar.1907s0001.1.p |
| *Capsella grandiflora* | CgTUG1 | Cagra.0483s0085.1.p |
|  | CgTUG2 | Cagra.1504s0064.1.p |
| *Capsella rubella* | CrTUG1 | Carub.0005s2834.1.p |
|  | CrTUG2 | Carub.0006s0452.1.p |
| *Crambe hispanica* | ChTUG1 | Crahi.0035s0022.1.p |
|  | ChTUG2 | Crahi.0054s0042.1.p |
| *Descurainia sophioides* | DsTUG1 | Desop.0015s0060.1.p |
|  | DsTUG2 | Desop.0229s0374.1.p |
| *Rorippa islandica* | RiTUG1 | Roisl.0016s0387.1.p |
|  | RiTUG2 | Roisl.0091s0792.1.p |
| *Eruca vesicaria* | EvTUG1 | Eruve.1184s0007.1.p |
|  | EvTUG2 | Eruve.1867s0002.1.p |
|  | EvTUG3 | Eruve.3686s0009.1.p |
| Arabidopsis thaliana | AtTUG1 | P38557 |
|  | AtTUG2 | P38558 |
| Arabidopsis lyrata | AlTUG1 | D7LSC6 |
|  | AlTUG2 | D7LYZ1 |
| *Camelina sativa* | CsTUG1-A | XP_010413372 |
|  | CsTUG1-B | XP_010469116 |
|  | CsTUG1-C | XP_010512522 |
|  | CsTUG2-A | XP_010423448 |
|  | CsTUG2-B | XP_010452470 |
|  | CsTUG2-C | XP_010491096 |
| *Chlamydomonas reinhardtii* | ChlRTUG | Q39582 |

* Phytozome, NCBI and UniProt IDs

**Evolutionary characterization of the identified α-, β- and γ-tubulin genes**


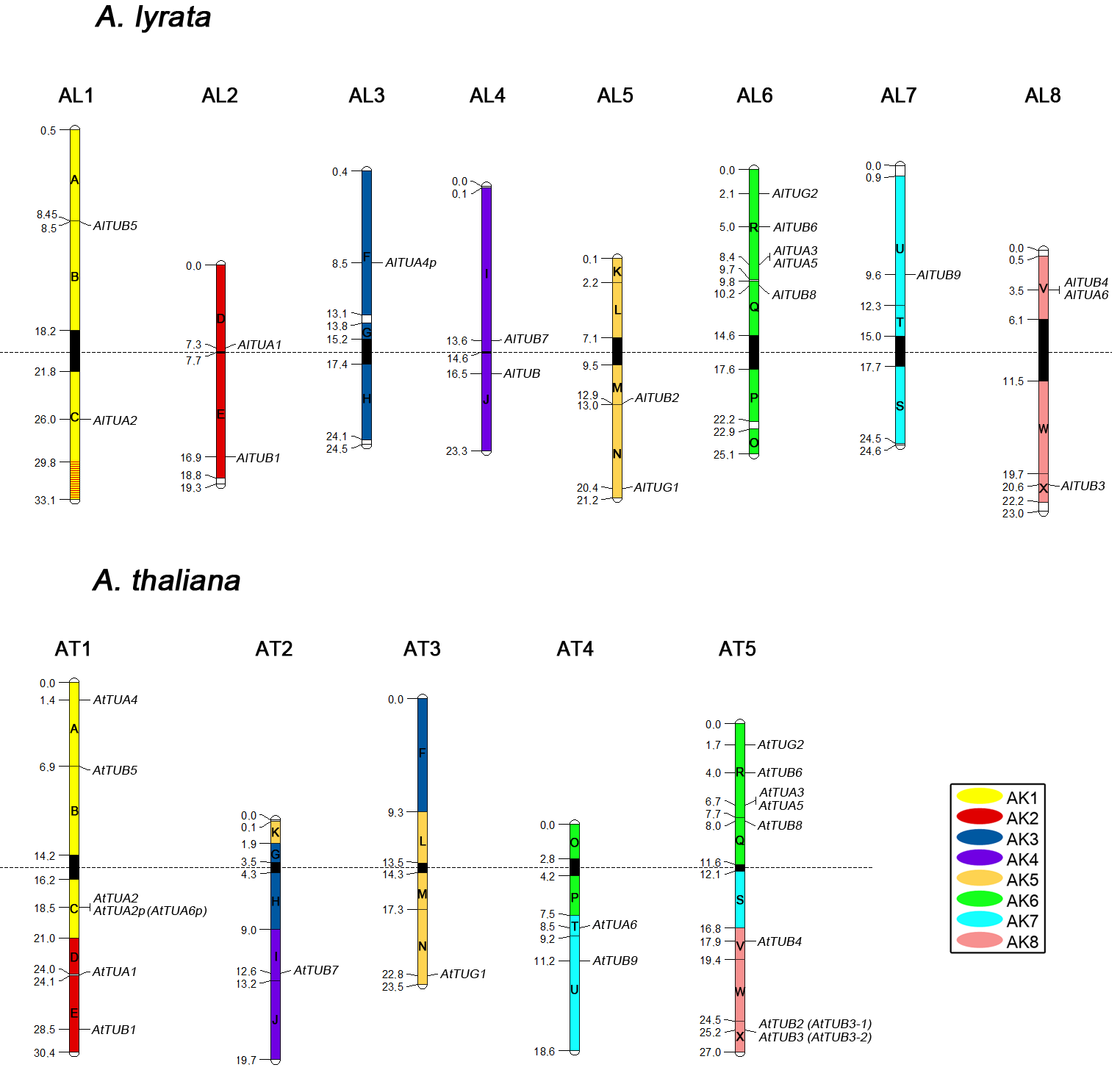


**Figure S10.** Ancestral crucifer karyotype (ACK) genomic blocks, mapped on *A. thaliana* and *A. lyrata* chromosomes with allocated tubulin genes. AK1-8 colors are referring to ancestral chromosomes, to which A-X blocks correspond. Centromeric regions are colored in black, while white indicate regions that were not assigned to specific ACK block.

**Table S7.** **Allocation of triplets of homeologous tubulin genes within certain ACK block among sub-genomes of *C. sativa***

| Sub-  family | ACK block | A (N^6^)  sub-genome | B (N^7^)  sub-genome | C (H^7^)  sub-genome | *A. thaliana* | *A. lyrata* |
| --- | --- | --- | --- | --- | --- | --- |
| α | D | *CsTUA1-A* | –^*^ | –^*^ | *AtTUA1*^†^ | *AlTUA1* |
|  | C | *CsTUA2-A* | *CsTUA2-B* | *CsTUA2-C* | *AtTUA2*  *AtTUA2p*^††^ | *AlTUA2* |
|  | R | *CsTUA3-A* | *CsTUA3-B* | *CsTUA3-C* | *AtTUA3* | *AlTUA3* |
|  | F | *CsTUA4-A* | *CsTUA4-B* | *CsTUA4-C* | – | *AlTUA4p* |
|  | A | – | – | – | *AtTUA4* | – |
|  | R | *CsTUA5p-A* | *CsTUA5-B* | *CsTUA5-C* | *AtTUA5* | *AlTUA5* |
|  | D | *CsTUA6-A* | *CsTUA6-B* | *CsTUA6-C* | – | – |
|  | V | – | – | – | – | *AlTUA6* |
|  | T | – | – | – | *AtTUA6* | – |
| β | E | *CsTUB1-A* | *CsTUB1-B* | –^**^ | *AtTUB1* | *AlTUB1* |
|  | C | – | – | *CsTUB1p-C^**^* | – | – |
|  | M | *CsTUB2-A* | *CsTUB2-B* | *CsTUB2-C* | – | *AlTUB2* |
|  | X | *CsTUB3-A* | *CsTUB3-B* | *CsTUB3-C* | *AtTUB3-1*^†††^  *AtTUB3-2*^†††^ | *AlTUB3* |
|  | X | – | – | *CsTUB3p-C*^***^ | – | – |
|  | V | *CsTUB4p-A* | *CsTUB4p-B* | *CsTUB4-C* | *AtTUB4* | *AlTUB4* |
|  | T | – | – | *CsTUB4p-C* | – | – |
|  | B | *CsTUB5-A* | *CsTUB5-B* | *CsTUB5-C* | *AtTUB5* | *AlTUB5* |
|  | R | *CsTUB6-A* | *CsTUB6-B* | *CsTUB6-C* | *AtTUB6* | *AlTUB6* |
|  | I | *CsTUB7-A* | *CsTUB7-B* | *CsTUB7-C* | *AtTUB7* | *AlTUB7* |
|  | Q | *CsTUB8-A* | *CsTUB8-B* | *CsTUB8p-C* | *AtTUB8* | *AlTUB8* |
|  | U | *CsTUB9-A* | *CsTUB9-B* | *CsTUB9-C* | *AtTUB9* | *AlTUB9* |
|  | U | *CsTUB9p-A^****^* | *CsTUB9p-B^****^* | – | – | – |
|  | J | *CsTUB-A* | *CsTUB-B* | *CsTUB-C* | – | *AlTUB* |
| γ | N | *CsTUG1-A* | *CsTUG1-B* | *CsTUG1-C* | *AtTUG1* | *AlTUG1* |
|  | R | *CsTUG2-A* | *CsTUG2-B* | *CsTUG2-C* | *AtTUG2* | *AlTUG2* |

* - possibly it could be *CsTUA1-Un* or other third homeolog, if such exists;

** - original gene most likely translocated (during shattering of ACK1 and ACK2 blocks), what was accompanied by pseudogenisation and origin of *CsTUB1p-C*;

*** - distantly located paralog of *CsTUB3-C*, probably, tandem duplicate;

**** - each of these genes appears to be ancient paralogs to *CsTUB9-A* or *CsTUB9-B,* between each other marked genes are homeologous;

^†^ Located 0.1Mbp outside of the D block;

^††^ Formerly named as *AtTUA6* or pseudogene – *AtTUB6p* (see Table S1);

^†††^ Formerly named as *AtTUB2* (*AtTUB3-1*) and *AtTUB3* (*AtTUB3-2*).


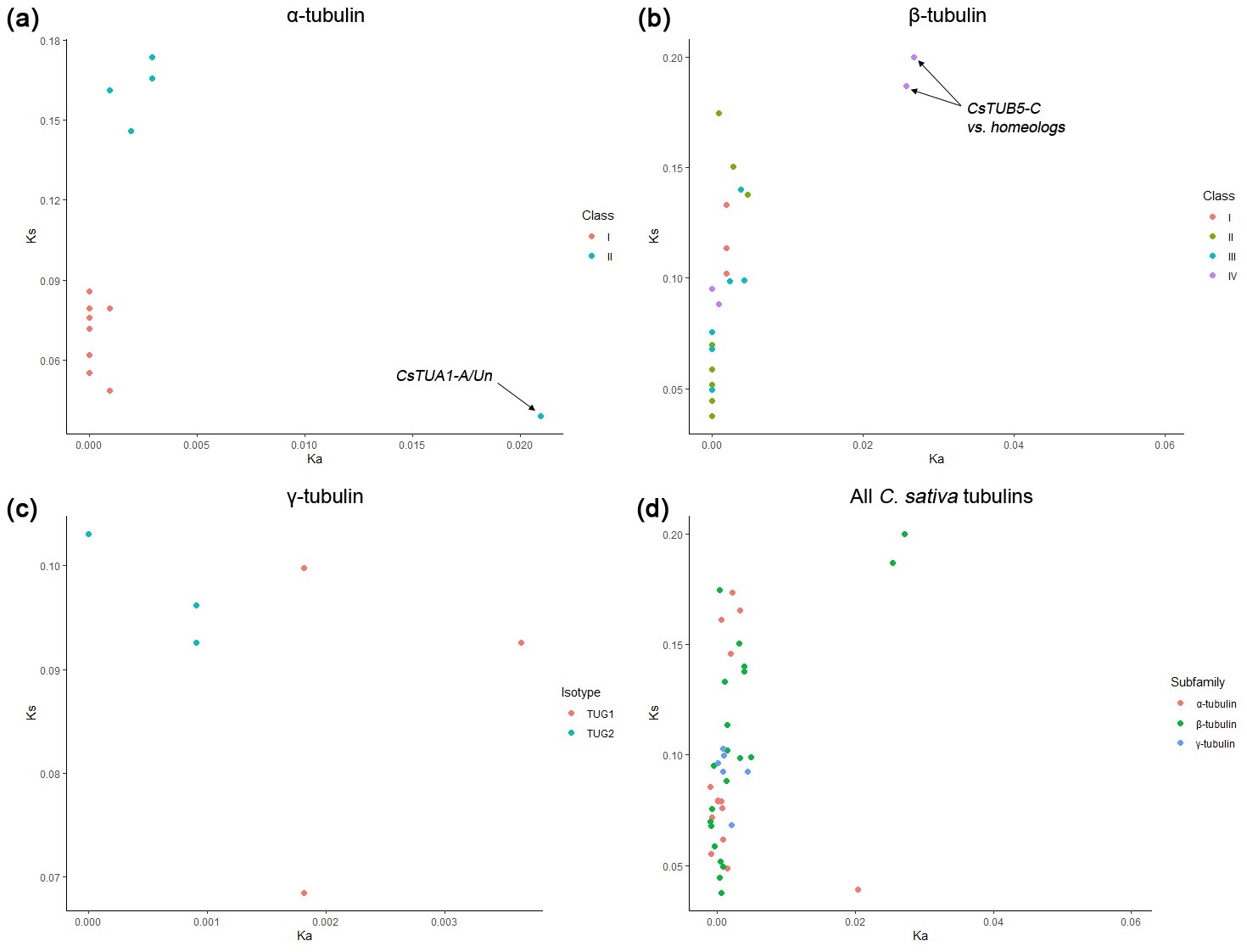


**Figure S11.** Dot plots representing *Ka* and *Ks* values for the identified functional gene homeologs of **(a)** α-tubulins, **(b)** β-tubulins, **(c)** γ-tubulins; and **(d)** the comparison of *Ka*-*Ks* values within different tubulin gene subfamilies.


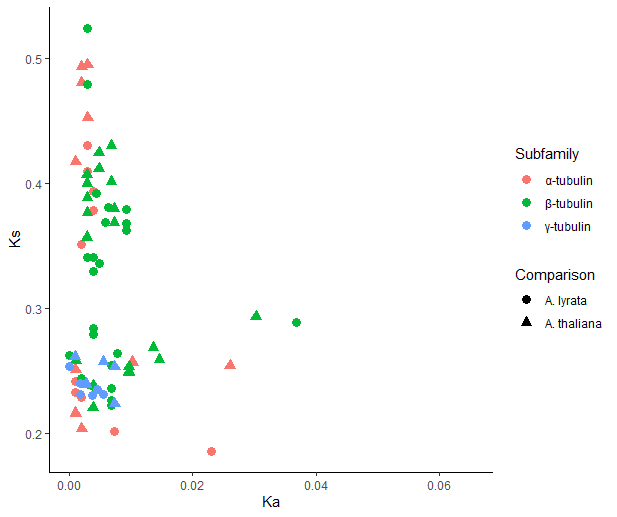


**Figure S12.** Dot plot representing *Ka* and *Ks* values for the identified functional tubulin genes in *C. sativa* compared to their orthologs (Table S6) in *A. thaliana* and *A. lyrata*.
